# Single-molecule fluorescence and cross-linking reveal ligand-gated Toc34 oligomerization dynamics

**DOI:** 10.1016/j.bpj.2026.02.006

**Published:** 2026-02-10

**Authors:** Sree Kavya Penneru, Sriram Tiruvadi-Krishnan, Rajan Lamichhane, Barry D. Bruce

**Affiliations:** 1 Biochemistry and Cellular & Molecular Biology, University of Tennessee, Knoxville, Tennessee 37916

## Abstract

Researchers have characterized the structural framework of the chloroplast TOC (translocon at the outer envelope membrane of chloroplasts) complex, but how this machinery orchestrates preprotein recognition and translocation at the molecular level remains unclear. Toc34, a membrane-anchored GTPase receptor within the TOC complex, plays a central regulatory role through its ability to oligomerize in a nucleotide-reversible, substrate-dependent manner. To investigate the dynamic behavior of Toc34, we employed single-molecule photobleaching, Förster resonance energy transfer (FRET), and chemical cross-linking to examine its oligomeric states under various conditions. Photobleaching revealed that Toc34 predominantly forms dimers in the basal state, but this dimerization is disrupted in the presence of the RuBisCO small subunit transit peptide and nonhydrolyzable GTP analogs (GMP-PNP, GTPγS), suggesting a conformational shift linked to precursor engagement and GTPase cycling. FRET analysis of GDP-bound Toc34 identified three conformational populations with distinct energy transfer efficiencies; no FRET signal was observed upon binding transit peptides/GTP analogs, indicative of increased conformational mobility or complete monomerization. Cross-linking data further support this oligomeric transition. These results demonstrate that Toc34 undergoes regulated conformational changes driven by nucleotide state and precursor binding, acting as a molecular switch that modulates TOC complex activity during chloroplast protein import. This study provides mechanistic insight into Toc34 ligand-gated conformational switching, revealing how dynamic GTPase interactions contribute to the fidelity and efficiency of chloroplast biogenesis and have broader implications for protein trafficking in plant organelles.

## Significance

The TOC-TIC system mediates the import of thousands of nuclear-encoded proteins into chloroplasts, essential for photosynthesis and plant growth. Toc34, a membrane-anchored GTPase receptor, regulates this gateway through oligomerization and nucleotide cycling. Using single-molecule photobleaching, FRET, and cross-linking, we directly visualized Toc34’s conformational heterogeneity and dynamic switching. With the fully monomeric fluorescent protein Green Lantern/mEGFP, we established a robust single-molecule signature of Toc34 dimerization and its regulation by transit peptides and nonhydrolyzable GTP analogs. Our findings reveal how nucleotide state and precursor engagement reshape Toc34’s oligomeric transitions, positioning it as a dynamic molecular switch in protein import. These results link structural flexibility to regulatory function, providing new insights that are unresolved by cryo-EM structures.

## Introduction

**Figure 1:**
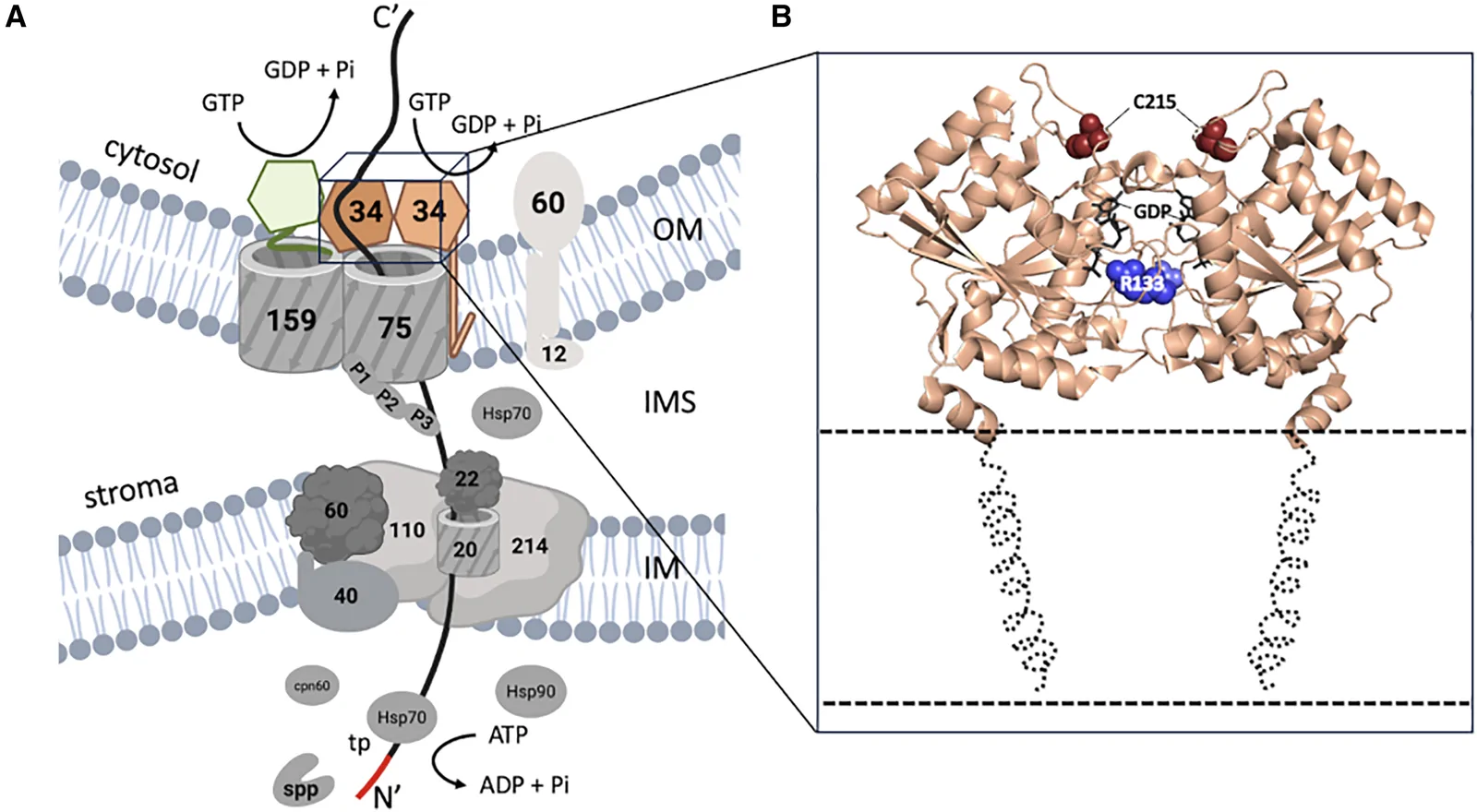
Structural illustration of chloroplast translocon and psToc34 homodimer. (*A*) Schematic representation of the chloroplast translocon, highlighting the TOC complex in the outer envelope membrane (OM) and the TIC complex in the inner membrane (IM), shown in the context of a bound preprotein. TOC and TIC subunits are shown in gray except for Toc34, shown in dark and light shades of beige for the left and right monomers, respectively. Toc34 contains a cytosolic GTPase domain and a single transmembrane helix anchoring it to the membrane. The GTPase domain of Toc159 is shown in green. The transit peptide (tp) is shown in red at the N-terminus of the preprotein. (*B*) Crystal structure of the *Pisum sativum* Toc34 homodimer (PDB: 1H65) (7) in the GDP-bound conformation. The R133 “finger” residues of the right and left monomers are shown in light and dark blue space-filling representation, respectively. The two bound GDP molecules are displayed in black. The C215 residues used in cross-linking assays are highlighted in maroon. The unresolved transmembrane helices are indicated as dotted helices, and the horizontal dotted line denotes the position of the membrane.

Plastids are ubiquitous organelles in plant cells, of which chloroplasts are a prominent subtype. In addition to driving photosynthesis, chloroplasts carry out critical metabolic processes, including the biosynthesis of amino acids and lipids, as well as nitrogen metabolism (1,2,3). Although chloroplasts are semiautonomous organelles, their genome encodes fewer than 125 proteins, meaning that the bulk of chloroplast proteins—estimated to be around 3600—are encoded by the nucleus, synthesized in the cytoplasm, and subsequently imported into the organelle (4,5). This import proceeds through posttranslational translocation across the outer and inner chloroplast envelope membranes via TOC (translocon at the outer envelope membrane of chloroplasts) and TIC (translocon at the inner envelope membrane of chloroplasts) translocons (6) (Fig. 1 *A*). Notably, the only currently available structure of the plant TOC complex is at 35-Å (8) resolution; however, the TOC/TIC supercomplex was recently resolved at 2.5 Å (9), yet the GTPase domains of the Toc34 subunits remain unresolved. However, none of these structures reveal how the translocons exhibit dynamic behavior or lateral movements modulated by the binding/interaction with transit peptides and nucleotide substrates (10).

The transport of proteins into the chloroplast depends on several membrane-bound components and associated chaperones. The channel-forming TOC complex is composed primarily of Toc34, Toc75, and Toc159, each encoded by a nuclear gene but localized to the chloroplast envelope (11). The transit peptide—an N-terminal signal sequence on precursor proteins—guides them to the TOC complex and is recognized by these membrane receptors (11,12). Such molecular interactions allow the precursor protein to pass through the channel in a regulated manner, relying on transient association and dissociation of the complex’s subunits (13). Toc34 and Toc159 can homodimerize and heterodimerize to facilitate translocation, requiring GTP hydrolysis and precise coordination of receptor and channel functions (14,15).

Central to this machinery is Toc34, a GTPase receptor that recognizes and translocates nuclear-encoded precursor proteins into chloroplasts. It consists of a G-domain, which contains a GTP-binding site, and a tail-anchor, an alpha-helical membrane domain that anchors the protein to the outer membrane (7). The G-domain cycles between GTP- and GDP-bound states, regulating interactions with preproteins, Toc159 and Toc75, thereby modulating the import machinery. So far, crystallographic studies (7,13,16,17) have only resolved the cytosolic G-domain of Toc34 in plants. The recent cryo-EM study from *Chlamydomonas* (9) has resolved the membrane domain but not the cytosolic G-domain. The tail-anchor is known to stabilize the protein in the membrane but has no direct influence on the GTPase activity or the oligomeric state of the protein (18). Site-directed mutagenesis studies underscore the significance of the P-loop (Walker A motif) and switch regions I/II in nucleotide binding and hydrolysis (17,19,20). Phosphorylation has also been shown to modulate Toc34’s GTPase cycle, altering oligomerization and import efficiency (10,17,19,20). Mutants of the *Arabidopsis* homolog at Toc33 highlight how phosphorylation and nucleotide cycling can fine-tune the recognition of precursor proteins (21).

A key structural motif in Toc34 is its arginine finger (e.g., R133 in *Pisum sativum* Toc34 and *Arabidopsis* Toc33), a hallmark of GTPase dimerization interfaces (22). This arginine residue inserts into the active site of the partnering monomer, stabilizing the transition state and promoting cooperative GTP hydrolysis (17,19). High-resolution crystallographic studies from both pea and *Arabidopsis* reveal that Toc34 can adopt distinct conformations (apo, GTP bound, and GDP bound) (7,13,16,17), with species-specific differences in loop flexibility and surface charge distribution (11,18). Chemical cross-linking agents such as disuccinimidyl suberate, glutaraldehyde, and zero-length crosslinkers have captured transient oligomeric states and mapped interfacial contacts with Toc159, Toc75, and transit peptides (23,24,25). These integrated structural and biochemical approaches have shaped a detailed functional model for Toc34’s essential role in protein import.

Although ensemble techniques (mutagenesis, crystallography, cross-linking) have revealed much about the Toc34 function, they average the heterogeneity and transient states intrinsic to GTPase-driven processes. Single-molecule fluorescence photobleaching can overcome these limitations by monitoring conformational changes in real time through the oligomeric state of Toc34 molecules. Observing changes in Toc34 under varying conditions of nucleotide and transit peptide directly captures how nucleotide cycling and dimerization influence its configuration and peptide binding. Compared with other G-proteins, Toc34 possibly acts as a gating mechanism through its homodimer dynamics, governing protein import across the chloroplast envelope. These findings bridge the gap between Toc34’s GTPase cycle stages, showing how the interplay of nucleotide binding and transit peptide engagement modulates import. Moreover, single-molecule Förster resonance energy transfer (FRET) experiments further elucidate the interactions between Toc34 and the transit peptide, ultimately expanding our understanding of the TOC complex in chloroplast biogenesis and plant biology.

## Materials and Methods

### Cloning and plasmid preparation

Fusion proteins were designed to incorporate fluorophores, specifically monomeric Green Lantern (mGL) (26) or monomeric enhanced green fluorescent protein (mEGFP) (27). For mGL-tagged fusion proteins, the fluorophore was linked to either the C- or N-terminus of 1–268 sequence length psToc34 (UniProt: Q41009) (28) via a short linker sequence (GGGGSAS). Additionally, mutant variants (R133A) were generated for both types, yielding four fusion proteins, each containing an 8X His-tag at the C-terminus. In case of mEGFP-tagged fusion proteins, only a single configuration was constructed, where mEGFP was N-terminally linked to Toc34 using the same short linker, with an 8X His-tag at the C-terminus, and its respective R133A mutant. All constructs were designed to be incorporated into the pET-29b (+) vector with kanamycin as a selection marker using SnapGene and codon-optimized for expression in *Escherichia coli* (*E. coli*) using the Twist Biosciences optimization interface. SS-tp construct was designed with a dual tag, N-terminal His-tag and an S-tag, and an additional C-terminal His-tag in a pET-30a (+) vector with kanamycin resistance. The construct was developed in our lab and has been characterized for its functionality (29).

### Structure predictions and energy minimization

Structural predictions were performed using the Phyre2 server, generating triplicate homology models for comparison. Phyre2 is an interface for template-based homology modeling. We used it to predict structures of fusion proteins based on available templates in the database. It is reliable in predicting structures with point mutations and can use multiple templates in building a homology model using the hidden Markov model (HMM). The predicted structures were further refined through energy minimization for 270 ps using NAnoscale Molecular Dynamics (NAMD 3.0), with force field parameters (CHARMM36) and molecular dynamics input files generated via CHARMM-GUI to obtain a stable structure. The resulting structures were assessed for stability and flexibility by aligning the models in Visual Molecular Dynamics and calculating the root-mean-square deviation (RMSD) of the Toc34 and linker residues. The optimized sequences were subsequently submitted to Twist Biosciences for synthesis in a pET-29b (+) vector with kanamycin resistance.

### Purification of fusion proteins and SS-tp

The plasmids were transformed into BL-21 (DE3) *E. coli* cells by heat shock. Transformed cells were then cultured in Luria-Bertani medium with 50 μg/mL kanamycin at 37°C. Protein expression was induced with 1 mM IPTG when the culture reached an optical density at 600 nm (OD600) of 0.4, followed by an additional 3 h of incubation. Cells were harvested by centrifugation at 5000 × *g*, and the resulting pellets were resuspended in lysis buffer (50 mM Tris, 150 mM NaCl (pH 7.5)) supplemented with protease inhibitors (100 μg/mL lysozyme, 1 mM PMSF, 2 μM leupeptin, 2 μM pepstatin). Lysis was performed using a microfluidizer at 15,000 psi. The cell lysate was then centrifuged at 12,000 rpm for 20 min, and the supernatant was incubated overnight with Co^2+^ affinity resin. The resin was washed with 20 column volumes of wash buffer (50 mM Tris, 150 mM NaCl (pH 7.5)), and the protein was eluted using elution buffer (50 mM Tris, 150 mM NaCl, 250 mM imidazole (pH 7.5)). The eluted protein was dialyzed against wash buffer to remove imidazole using a Mini Slide-A-Lyzer (Thermo Scientific) with a 10K MCWO cutoff. The protein concentration was determined by measuring the sample absorbance at 562 nm using the Pierce BCA Protein Assay. For purification of SS-tp, a protocol similar to that for the fusion protein was followed to transform BL-21(DE3) *E. coli* cells. The transformed bacteria were induced with 1 mM IPTG at an OD of 0.4 and pelleted by centrifugation at 5000 × *g* after 3 h of expression. The protein was purified under denaturing conditions, starting with lysis by resuspending the cell pellet in lysis buffer (50 mM Tris, 150 mM NaCl, 6M urea (pH 8.0)) supplemented with protease inhibitors (100 μg/mL lysozyme, 1 mM PMSF, 2 μM leupeptin, 2 μM pepstatin) and using a microfluidizer at 15,000 psi for three cycles. The lysate was incubated with Co^2+^ affinity resin overnight in a cold room, followed by washing with wash buffer (50 mM Tris, 150 mM NaCl, 6 M urea (pH 8.0)) for 20 column volumes. Elution was performed with elution buffer (50 mM Tris, 150 mM NaCl, 250 mM imidazole, 6M urea (pH 8.0)). Eluates were dialyzed using a Mini Slide-A-Lyzer (Thermo Scientific) with a 3K MWCO cutoff in wash buffer without urea and then stored at −80°C.

### Low-temperature fluorescence spectroscopy of fusion proteins

The excitation and emission spectra of fusion proteins were measured using a Photon Technology International fluorimeter. Approximately 300 μL of a 1 mg/mL sample was loaded into an EPR tube, and the tubes were gradually frozen in liquid nitrogen. The frozen tubes were wiped with a Kimwipe to remove any residual water formed due to condensation before being placed in the sample holder filled with liquid N_2_. A scan was taken from 300 nm to 700 nm with a 1-mm aperture size and an integration time of 0.5 milliseconds. For emission spectra, the sample was excited at 488 nm. An average of three readings was taken and normalized to plot the final spectrum of excitation and emission scans of all the fusion proteins.

### Cross-linking of fusion proteins

A 15 μM fusion protein sample was reduced with 100 μM tris(2-carboxyethyl)phosphine (TCEP) for 1 h in the dark at room temperature (RT). After reduction, the sample was desalted using Zeba spin columns, which were spun at 1000 × *g* for 2 min on a tabletop centrifuge. Subsequently, 100 μM bismaleimidohexane (BMH) cross-linker with a 13-Å spacer was added, and the reaction mixture was incubated for 30 min in the dark at RT to facilitate cross-linking. To study the influence of nucleotides and the transit peptide, 150 μM of each variant was incubated separately with reduced protein for 30 min before cross-linking. The reaction was quenched with 0.1 μM β-mercaptoethanol, and the samples were prepared for SDS-PAGE by reducing them in 5X reducing buffer (250 mM Tris-HCl (pH 6.8), 500 mM DTT, 10% w/v SDS, 0.025% bromophenol blue, and 25% w/v glycerol).

### Immunoblotting to characterize fusion proteins and detect Toc34 dimers

To characterize fusion proteins, a 10%–20% Tris-Tricine SDS-PAGE was run at a constant 70 V for the first 20 min and 100 V for the next 2 h or until the dye front reached the end of the gel. Subsequently, the gel was transferred to a polyvinylidene fluoride membrane at 50 V for 2 h. The transferred blot was incubated in blocking buffer (5% nonfat milk) for 1 h at RT and then washed with tris-buffered saline with Tween-20 (TBST) (50 mM Tris, 150 mM NaCl (pH 7.5), 0.1% Tween-20). Then, the blot was incubated with an in-house polyclonal primary antibody raised in rabbit against psToc34 (1:50,000 in blocking buffer) overnight at 4°C. Then, the blot was washed with TBST three times, each for 10 min. After washing, the blot was incubated with the horseradish peroxidase conjugated goat antirabbit secondary antibody (1:50,000 titer in blocking buffer) for an hour at RT. The blot was washed three to four times with TBST for 5 min each. For detection, the blot was treated with a 1:1 mixture of luminol and peroxide to visualize the protein signal through chemiluminescence on a Bio-Rad imaging system. The bands were quantified using the Image Lab software provided by Bio-Rad. The volume of each band is measured by assigning a constant box size to each band and background-subtracting with the average of the same box across three different spots on the blot. Percent dimer signal (%*d*) was estimated by $\%\;d=\frac{d}{\left(d+m\right)}\times 100$, where *d* is the signal from the dimer band, and *m* is the signal from the monomer band, and percent monomer signal (%*m*) was estimated by $\%\;m=\frac{m}{\left(d+m\right)}\times 100$. Bar plots were generated from three replicated blots, and a two-way ANOVA was performed in GraphPad Prism to assess statistical significance.

### Site-specific labeling of Toc34

20 μM of Toc34 protein is reduced with a 10-fold molar excess of TCEP for an hour at RT and then desalted using Zeba spin columns to remove excess TCEP. The reduced protein is then incubated overnight in the dark at 4°C with a 10-fold molar excess of Alexa Fluor 555 maleimide for the donor and Alexa Fluor 647 maleimide for the acceptor. The sample is then passed through Zeba desalting spin columns to remove excess dye. The absorbance of the labeled protein is then measured at its maximum to determine the labeling efficiency, along with that of the dye.

### Single-molecule total internal reflection fluorescence microscopy

**Figure 2:**
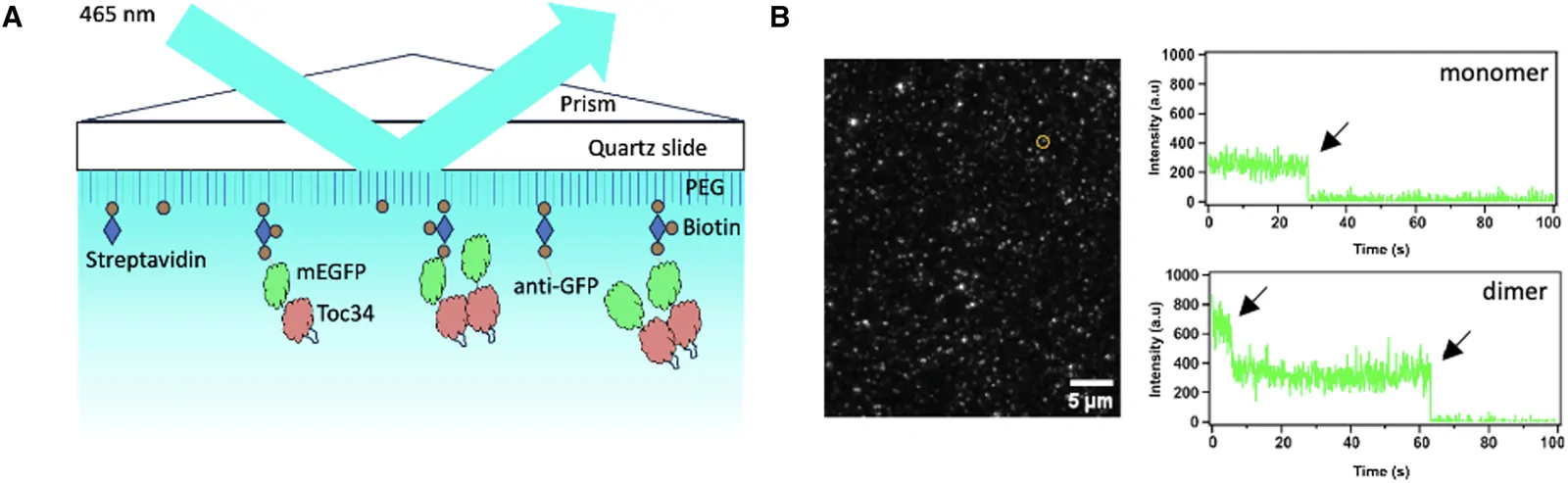
Illustration of TIRF microscopy and representative single-molecule fluorescence trajectories. (*A*) Illustration of prism TIRF with the fusion protein immobilized on the quartz slide with biotinylated anti-GFP antibody bound to streptavidin. (*B*) Left: TIRF microscopy image showing surface-immobilized Toc-34 mEGFP fusion proteins. Each fluorescent spot corresponds to an individual Toc34 protein or protein complex. Right: representative single-molecule fluorescence intensity time trajectories of Toc34-mEGFP. The top panel shows a single-step photobleaching event representing a monomer, whereas the bottom panel shows a two-step photobleaching trace indicating a dimeric Toc34 complex.

For single-molecule total internal reflection fluorescence microscopy, we used an imaging buffer containing 50 mM HEPES (pH 7.5), 150 mM sodium chloride, and 2 mM Trolox. 15 μM purified fusion protein was reduced with 100 μM TCEP for an hour, followed by desalting to remove excess TCEP, and then incubated with 150 μM of SS-tp for 30 min (or with 150 μM of nucleotides). The various conditions tested were wild-type (WT) fusion protein in the presence of 1) SS-tp, 2) GTP, 3) GMP-PNP, 4) GTPγS, 5) reduced carboxymethylated α-lactalbumin (RCMLa), an irrelevant (noncognate) disordered polypeptide control, and 6) WT by itself. The mutant R133A was used as another control for homodimer formation. These samples were diluted to approximately 500 pM in the imaging buffer before being loaded onto a slide. The quartz slides and coverslips were cleaned and passivated with a 20:1 mix of polyethylene glycol (PEG) and biotinylated PEG, as previously described (30,31). A sample chamber was prepared using PEGylated slides and coverslips, secured with double-sided tape and grease, and coated with 0.2 mg/mL streptavidin. The sample chamber was washed with imaging buffer to remove unbound streptavidin molecules. For photobleaching experiments, the biotinylated anti-GFP antibody was diluted in the imaging buffer containing 0.05% BSA to a final concentration of 20 nM. The antibody solution was then injected into the sample chamber and incubated for 20 min, followed by a wash step. Then, the diluted protein sample was injected into the chamber and incubated for 30 min, followed by another wash step. The chamber was rinsed with a protocatechuate dioxygenase and protocatechuic acid oxygen-scavenging system in imaging buffer (32). The slide was excited using a 465-nm laser (Fig. 2 *A*). For single-molecule Förster resonance energy transfer (smFRET) measurements, slides were prepared by immobilizing 2 nM Alexa 555-labeled Toc34 (donor) via a 20-min incubation with biotinylated NTA antibodies, followed by two washes with image buffer. 2 nM of a 6X histidine peptide was applied to the slide and incubated for 15 min to saturate all free NTA. The slide was then washed twice, and a fourfold molar excess of Alexa 647-labeled Toc34 diluted in the oxygen-scavenging system was injected onto it. After a 20-min incubation, the slide was imaged under excitation at 532 nm (Fig. 6 *A*). The emission intensities were collected on an EMCCD camera (Andor Technology) with 100-ms integration time using a custom single-molecule data acquisition program (30,33). Single-molecule time trajectories were extracted using scripts written in IDL software (Harris Geospatial Solutions). The data acquisition and extraction packages were downloaded from Dr. Taekjip Ha’s laboratory (https://github.com/Ha-SingleMoleculeLab).

### Single-molecule photobleaching data analysis

The single-molecule mEGFP fluorescence photobleaching trajectories were manually analyzed using custom-built MATLAB code to record the bleaching steps corresponding to the number of mEGFP molecules held together by the toc34 proteins (31,34,35). A single-step photobleaching corresponds to the monomeric form, and a double-step photobleaching with double signal intensity corresponds to the dimeric form of Toc34. To account for the nonmature fluorescent proteins in the sample, we used a maturation efficiency of 0.7, as suggested (36), and a Bayesian method to correct the stoichiometry (35,37,38). With the maturation correction, the quantified percent values of monomers and dimers are plotted as a bar plot. In addition to the manual analysis of fusion proteins with mEGFP, another MATLAB code was developed to test an automated analysis method (https://github.com/sreekavyapenneru/Single-molecule), which detected monomer and dimer traces based on their signal intensities. The average of the signal within the first 1500 milliseconds was compared with the average after 45,000 milliseconds (the last 50 frames) after complete photobleaching. The average intensity after complete photobleaching of the fluorophore was used for background subtraction, and then the values were normalized to the intensities of a monomer (average intensity of monomer ∼200). The normalized values were plotted as a histogram and were fitted to a Gaussian distribution. We obtained two distributions: the area under the first peak, with low signal intensity, is considered the monomer population, and the second peak, with almost twice the signal intensity of the monomer, is considered the dimer population. The percent dimer population is estimated by dividing the area under the second peak by the total area under both peaks and then multiplying by 100; the percent monomers is estimated similarly.

To estimate uncertainty in the percent monomer or dimer population, we performed propagation of errors for the function (*f*) involving covariance between monomer (*m*) and dimer (*d*) population (*σ*_*ⅆm*_) (39). An example of determining uncertainty in the dimer population is shown below:

Function (*f*):

(1) $$\%\;d=\frac{d}{\left(d+m\right)}\times 100$$

Propagation of errors:

(2) $${\sigma_{f}}^{2}={\left(\frac{\partial f}{\partial d}\right)}^{2}{\sigma_{d}}^{2}+{\left(\frac{\partial f}{\partial m}\right)}^{2}{\sigma_{m}}^{2}+2\left(\frac{\partial f}{\partial d}\cdot \frac{\partial f}{\partial m}\cdot \sigma_{\mathrm{d}m}\right)$$

Standard deviation ($\sigma_{f})$:

(3) $$\sigma_{f}=\sqrt{\frac{\sum {\sigma_{f}}^{2}}{n}}$$

Equation 1 was used to calculate the percent dimer population, Equation 2 was used to calculate the error in percent dimer population, and the standard deviation was calculated using Equation 3.

### Single-molecule FRET data analysis

smFRET data were analyzed following previously established protocols (40,41). The trajectories acquired from the TIRF microscope were manually analyzed using custom code written in MATLAB. Donor and acceptor intensity trajectories were background-subtracted and corrected for donor leakage into the acceptor channel. FRET efficiency (E) was then calculated using the formula E = IA/(ID + IA), where ID and IA are the corrected donor and acceptor intensities, respectively. Only trajectories exhibiting anticorrelated donor and acceptor emission and single-step photobleaching were selected for further analysis. FRET traces were binned to generate individual histograms, which were then compiled into a composite histogram from multiple trajectories. Histograms were fitted with Gaussian functions using multipeak analysis in OriginPro 2024b. Furthermore, FRET time traces were fitted with an HMM, using a program previously described (42). A transition density plot (TDP) was constructed from the dynamic population processed in MATLAB R2024b. The resulting fitted population was used to generate the TDP and define cutoff values. These cutoff values were subsequently applied to calculate state transitions (e.g., state 1 to state 2, state 1 to state 3, etc.). Dwell times for each transition were plotted as histograms, with bin widths determined by the Freedman-Diaconis rule. Each histogram was then fitted with a monoexponential decay function, and the amplitude of the fit provided the number of transitions, and the slope provided the corresponding rate constant. Goodness of fit was assessed using the reduced chi-squared test. Histogram generation, curve fitting, and chi-squared evaluations of dwell time analysis were conducted using Igor Pro 8.04. We then calculated the equilibrium rate constant (*k*_*eq*_), defined as the ratio of the average forward rate constant to the average reverse rate constant (Figs. 8 *D* and S15).

### Peptide sequence features and Toc34 recognition score

To quantify the propensity of peptide sequences to be recognized by Toc34, we developed a custom computational pipeline implemented in Python. Input peptide sequences were provided in plain-text format and converted to numerical representations of their biochemical properties, including a score for each positive, negative, aromatic, and glycine/proline residues. Sliding-window averaging (window equals five residues) was used to capture local sequence environments relevant to Toc34 interaction. For each sequence, a Toc34 recognition score was computed by summing weighted contributions of features present in canonical transit peptide sequences and divided by the features absent. The weights provided were tailored to highlight the FGLK domains of the transit peptide that are crucial for recognition by Toc34 (29), and a plus one is added in the denominator to avoid dividing a value with zero, as shown in Equation 4. For visualization, feature profiles—including charge, aromaticity, disorder (G/P), and the final recognition score—were plotted using Python’s Matplotlib. The complete code is available on GitHub (https://github.com/sreekavyapenneru/Single-molecule).

(4) $$score=\frac{\left(3*aromatic+positive+3*GP-rich\right)}{\left(1+3*negative\right)}$$

## Results

### Monomeric fluorescent proteins act as direct reporters of the Toc34 oligomeric state

Although the structure of Toc34 in the membrane or as part of the TOC translocon from plant systems is yet to be resolved, the available crystal structure of psToc34 (PDB: 1h65) (7) and cryo-EM structure of TOC-TIC from *Chlamydomonas* (PDB: 7XZI) (43) show differences in the oligomeric state of Toc34. As previous studies (7,8,10,16,23,44,45) on plant systems support the presence of oligomeric forms of Toc34, we expect a different organization of the TOC-TIC complex in plants compared with algal systems. In our lab, we study pea plants as a model organism to elucidate the TOC-TIC complex, so we considered the psToc34 sequence for all our studies here. In Fig. 1 *A*, we show a model TOC-TIC complex in plants, where the G-domain of Toc34 (dark beige) oligomerizes with another G-domain of Toc34 (light beige) to form a homodimer, which acts as a receptor for transit peptide or oligomerizes with the G-domain of Toc159 (green) to form a heterodimer, facilitating its interactions with Toc75, involved in protein import. The crystal structure (PDB: 1h65) (7) alone demonstrates that the conserved arginine 133 forms an arginine finger that is responsible for dimer formation, and the conserved cysteine does not participate in the dimeric interface (Fig. S1). The dimer structure, shown in Fig. 1 *B*, is in a GDP bound state, with two conserved arginine residues that form an “arginine finger” spanning the dimeric interface, and lacks the membrane domain at its C-terminus. The structure also indicates that the conserved cysteine is surface exposed and can therefore be covalently labeled with a maleimide-functionalized dye. We initially exploited this single cysteine to conjugate an Alexa 488 fluorophore for single-particle TIRF imaging (Fig. S2). Unfortunately, the labeling efficiency was low and highly variable, preventing accurate quantification of the dimer/monomer ratio. Nonetheless, both monomeric and dimeric populations were clearly observed using the purified and labeled protein obtained from this approach.

To overcome this, we directly fused Toc34 to two different fluorescent reporter proteins, mGL and mEGFP, and we tested them using single-molecule photobleaching experiments. Unlike small-molecule fluorophores, which often exhibit variable labeling stoichiometry or quenching, fluorescent proteins can provide more consistent fluorescence when genetically fused. Moreover, mGL provides several advantages, including an exclusively monomeric form, high quantum yield, and superior photostability with minimal photobleaching (26). Initially, we designed both N- and C-terminal fusions of Toc34 ΔTM with mGL, using either a long or a short linker sequence. To assess structural integrity and dimerization potential, we used Phyre2 (46) to generate homology models of the fusion proteins (Fig. S3) and compared their RMSDs. The construct with a short linker (GSGSGS) yielded an RMSD of 1.5 Å, whereas the more extended linker sequence (GGSDGGSDGGSDGGSD) yielded 3.1 Å, indicating greater flexibility. A short linker sequence was selected to enhance stability and minimize steric hindrance between the fusion partners. The fusion proteins have been characterized by SDS and Western blots for their size and in-gel fluorescence (Fig. S4). For single-molecule studies, we immobilized the fusion protein on a quartz slide using an anti-GFP antibody for specific binding (Fig. S5). We excited the sample with a 465-nm laser (Fig. 2 *A*). We observed single-step and two-step photobleaching, corresponding to monomer and dimer Toc34, respectively (Fig. 2 *B*).

### Toc34 is stabilized as a homodimer in its GDP-bound form

Although mGL and mEGFP have broadly similar excitation and emission profiles (Fig. S6), mGL is reported to have a considerably shorter photobleaching half-life, approximately 30.8 s, compared with mEGFP with 239 s (26) (Table S1). This enhanced photostability can be advantageous for extended or in vivo imaging applications. However, in our single-molecule TIRF experiments, mGL-fused Toc34 displayed a gradual fading over a 2-min acquisition window (Fig. S7, *A*–*C*), unlike the more distinct photobleaching events observed for Alexa 488-labeled Toc34 and mEGFP-fused Toc34 (Figs. S2–S7, *D*–*F*). Despite this gradual decay, the initial fluorescent intensity of each particle could be approximated by subtracting its end-of-interval fluorescence from its start-of-interval fluorescence. By examining 20 representative traces for putative dimers and monomers, we established that this differential measurement effectively reports on the oligomeric state of Toc34-mGL complexes in single-particle TIRF imaging.

**Figure 3:**
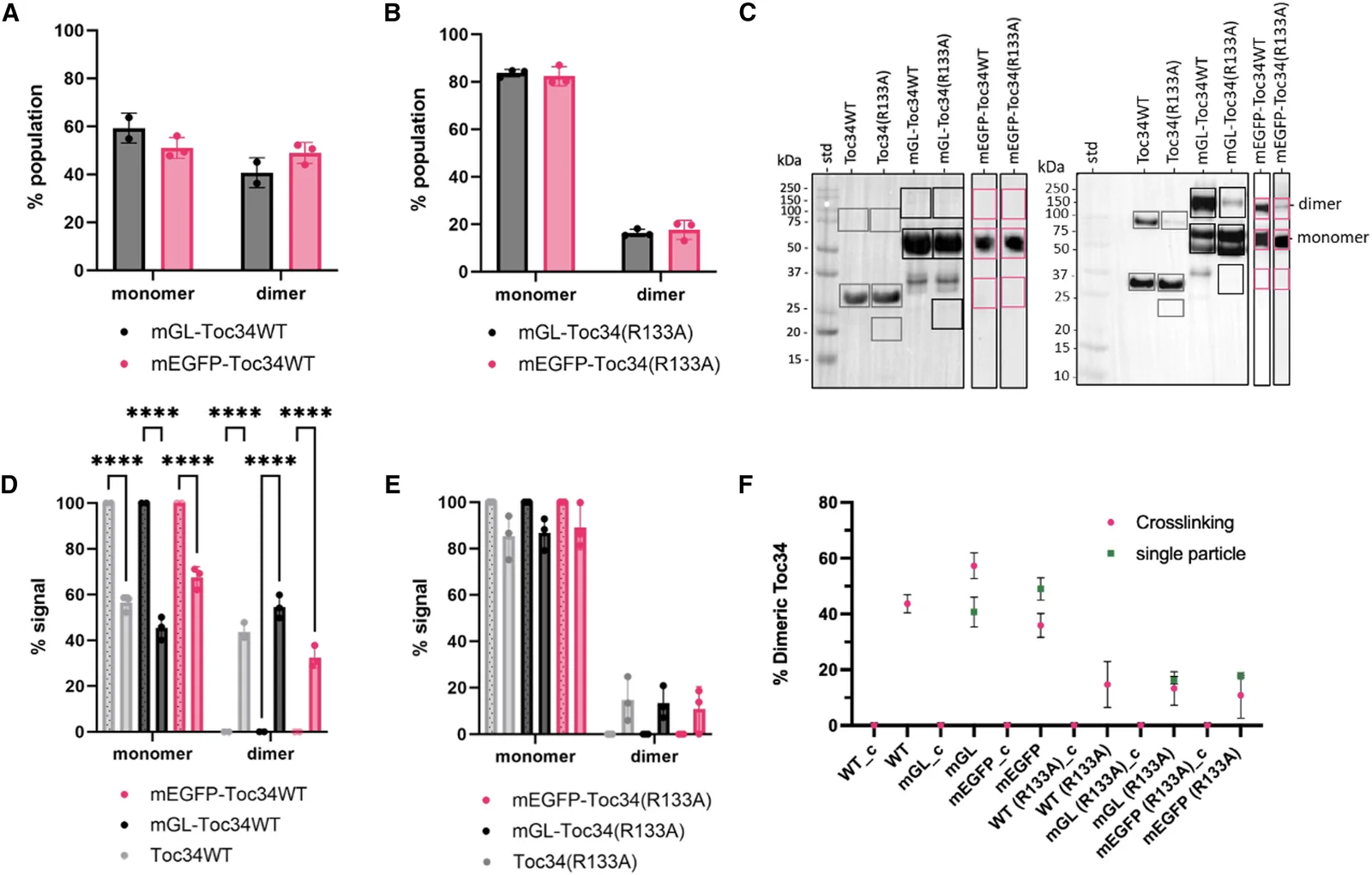
Quantifying the oligomeric state of Toc34WT and R133A. (*A*) A bar plot of quantified fluorescent time traces of monomer and dimer populations of mGL-Toc34WT in dark gray and the mEGFP-Toc34WT sample in pink. (*B*) A bar plot of quantified fluorescent time traces of monomer and dimer populations of mGL-Toc34(R133A) in dark gray and mEGFP-Toc34(R133A) in pink. (*C*) Immunoblot of samples before cross-linking on the left and after cross-linking with BMH on the right, showing nonfusion Toc34WT, R133A in the first two lanes, followed by mGL and mEGFP fusion proteins with their respective R133A mutants. The monomer and dimer bands are highlighted in boxes that are used for quantification in ImageLab software. The empty boxes at the bottom are used for background subtraction. (*D*) A bar plot of WT monomer and dimer population quantified from the blots in (*C*). The bars with a dotted pattern are from samples before cross-linking, and the bars without a dotted pattern are after cross-linking. (*E*) A bar plot of R133A monomer and dimer population quantified from the blots in (*C*). The bars with a dotted pattern are from samples before cross-linking, and the bars without a dotted pattern are after cross-linking. (*F*) Scatter plot comparing the dimer population obtained by cross-linking and single-particle analysis. The sample name with _c on the x-axis corresponds to before cross-linking, and the other is after cross-linking. Bar plots are made with data quantified from replicate experiments, and the error bars represent 95% confidence intervals generated in GraphPad Prism. *p*-values are obtained through two-way ANOVA analysis. ^∗^, *p* ≤ 0.05; ^∗∗^, *p* ≤ 0.01; ^∗∗∗^, *p* ≤ 0.001; ^∗∗∗∗^, *p* ≤ 0.0001.

These observations become particularly meaningful when considering that Toc34’s best-characterized form is a GDP-bound dimer, as captured in the crystal structure (PDB: 1h65). Indeed, our single-molecule fluorescence measurements revealed that 40.7% ± 5.4% of mGL-Toc34WT molecules adopted a dimeric form, whereas 59.3% ± 5.4% were monomeric (Fig. 3 *A*). In contrast, mEGFP-Toc34WT showed 51.0% ± 6.0% monomers and 49% ± 6.0% dimers. The essential function of the conserved arginine finger (Arg133) in the dimer interface was highlighted by R133A mutants. Both mGL-Toc34(R133A) and mEGFP-Toc34(R133A) samples exhibited dramatically reduced dimer populations (16.3% ± 3.0% and 17.6% ± 5.0%), underscoring the structural importance of Arg133 for stable homodimer formation (Fig. 3 *B*). To further confirm the dimerization potential of the ΔTM Toc34-mGL constructs, we subjected all four fusion variants (N- and C-terminal fusions, each in duplicate) to cross-linking assays with BMH (bismaleimidohexane). In each case, we detected a dimeric species, indicating that the C-terminal fusion to a large fluorescent protein does not abolish dimer formation (Fig. 3 *C*, right) compared with the controls without cross-linking (Fig. 3 *C*, left). The doublets in our blots with cross-linking are expected to be caused by a truncated fraction of the protein/proteolytically cleaved over time. Though observed on the blot, this fraction will not be detectable in our single-molecule experiments without a functioning fluorophore. Cross-linking assays corroborated these trends, suggesting that mGL-Toc34WT maintained a dimerization trend of 57.3% ± 4.6% (Fig. 3 *D*). The mGL and mEGFP R133A mutants showed a decrease in dimer population, to 13.3% ± 6.0% and 10.8% ± 8.2%, respectively (Fig. 3 *E*). Our R133A mutants with a His-tag do not form dimers, confirming that the His-tag is not involved in dimer formation. Notably, even the nonfused Toc34WT showed a comparable dimer fraction (43.7% ± 3.3%; Fig. 3 *F*). These data strongly suggest that tagging Toc34 with mGL—or leaving it untagged—does not substantially disrupt the native equilibrium between dimeric and monomeric states.

Interestingly, mEGFP-Toc34WT yielded a notably lower dimer fraction (35.9% ± 4.3%) in cross-linking assays compared with the mGL fusion protein (57.3% ± 4.6%) (Table S2). Sequence analysis identified several C-terminal substitutions (L232H, G233D, F224R, and L222K) present in mGL but absent in mEGFP, likely rendering mGL more conformationally flexible. This plasticity may increase degrees of freedom in dimer formation in the mGL-fused construct. Although slight variations in absolute dimer percentages were observed across different methodologies, the overall consistency in relative distributions suggests that the choice of fluorophore can meaningfully influence how we track Toc34 oligomerization.

Taken together, these findings reinforce the conclusion that GDP binding strongly favors Toc34 dimerization, consistent with the crystal structure’s snapshot of the GDP-bound state (PDB: 1h65) (7). The R133A results confirm that a properly configured arginine finger is crucial to maintaining this dimeric assembly. Although both mGL and mEGFP fusions can report on Toc34 oligomerization, their distinct photophysical properties—in particular, mGL’s slower photobleaching and higher flexibility—can yield different levels of dimer detection. This highlights the importance of choosing an optimal fluorescent label when investigating the dynamic equilibria of GTPases. Moreover, the sensitivity of Toc34 dimerization to nucleotide state, coupled with the significant effect of even a single amino acid change, suggests that fine-tuning the dimer interface may be a critical regulatory mechanism in the broader TOC translocon machinery.

### Nonhydrolyzing nucleotides destabilize Toc34 homodimers

A central feature of Toc34’s GTPase mechanism is its ability to shuttle between GDP- and GTP-bound states, adopting distinct oligomeric conformations in each. Building on our earlier findings that Toc34 forms a stable dimer in the GDP-bound conformation (Fig. 3), we assessed the impact of two nonhydrolyzing GTP analogs, GMP-PNP and GTPγS. These analogs mimic Toc34’s GTP-bound “transition” state, thereby offering insight into the intermediate stages of the GTPase cycle. Importantly, Mg^2+^ was provided at a 1:1 ratio with each nucleotide, as Mg^2+^ is required for stable nucleotide association.

**Figure 4:**
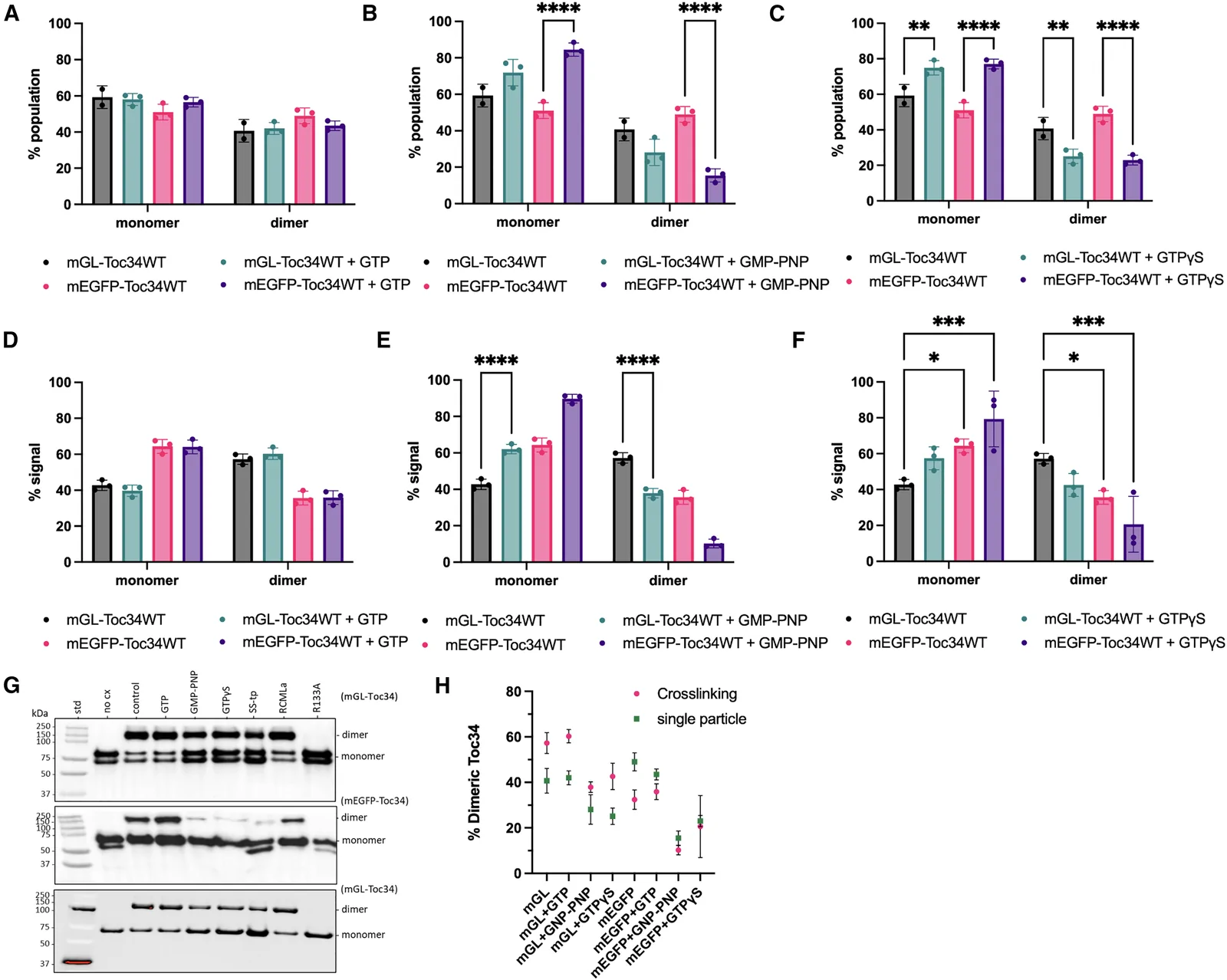
Influence of nucleotides on Toc34 fusion protein. (*A*) Bar plot made with quantified single-molecule data in the presence of 150 μM GTP. (*A–F*) mGL-Toc34WT is in dark gray as a control, and mEGFP-Toc34WT is in pink as a control. mGL and mEGFP fusion proteins in the presence of nucleotide are in green and purple, respectively. (*B*) is in the presence of 150 μM nonhydrolyzing nucleotide, GMP-PNP. (*C*) is in the presence of 150 μM slowly hydrolyzing nucleotide, GTPγS. (*D*) Bar plot from quantifying immunoblots of cross-linked Toc34 fusion protein in the presence of 150 μM GTP. (*E*) is in the presence of 150 μM nonhydrolyzable nucleotide, GMP-PNP, and (*F*) is in the presence of 150 μM, slowly hydrolyzing nucleotide, GTPγS. (*G*) Immunoblot of mGL-Toc34WT protein cross-linked with BMH, against Toc34 antibody, first lane after standard protein with no BMH; second lane, control; third lane, with 150 μM GTP; fourth lane, 150 μM GMP-PNP; fifth lane, GTPγS; sixth lane, with 150 μM SS-tp; seventh lane, with 150 μM reduced carboxymethylated lactalbumin (RCMLa); and eighth lane, mutant R133A protein, in the top panel. Immunoblot of mEGFP-Toc34WT samples against Toc34 antibody in the middle panel and stain-free imaged blot of cross-linked mGL-Toc34WT samples in the bottom panel. (*H*) Scatter plot comparing the dimer population obtained by cross-linking and single-particle analysis. Bar plots are made with data quantified from replicate experiments, and the error bars represent 95% confidence intervals generated in GraphPad Prism. *p*-values are from two-way ANOVA analysis. ^∗^, *p* ≤ 0.05; ^∗∗^, *p* ≤ 0.01; ^∗∗∗^, *p* ≤ 0.001; ^∗∗∗∗^, *p* ≤ 0.0001.

To probe the role of nucleotides in stabilizing these assembly states, we performed control experiments with GTP—a hydrolyzable nucleotide—which yielded 42.0% ± 3.0% and 43.5% ± 2.4% dimer populations for the WT mGL and mEGFP fusion proteins, respectively, in single-molecule studies (Fig. 4 *A*). This suggests that GTP hydrolysis drives Toc34 away from the fully dimeric (GDP-like) conformation, reflecting a dynamic equilibrium consistent with other GTPase-based systems. Our single-molecule measurements of mGL-Toc34WT in the presence of GMP-PNP revealed 28.1% ± 6.5% dimers and 15.5% ± 3.1% dimers for the mEGFP fusion protein (Fig. 4 *B*). In the presence of GTPγS, we observe 25.1% ± 3.6% dimers for the mGL fusion protein and 23.0% ± 2.5% dimers for the mEGFP fusion protein. (Fig. 4 *C*). These results extend our earlier observations of potential steric or conformational influences introduced by different fluorescent tags. Cross-linking data corroborated this finding, showing 57.3% ± 4.6% and 35.9% ± 3.5% dimers for control WT mGL and mEGFP fusion proteins (Fig. 4 *D*) and 37.9% ± 2.4% dimers for mGL fusion protein in the presence of GMP-PNP and 10.3% ± 2.0% dimers for mEGFP fusion proteins (Fig. 4 *E*). The flexibility between the two fluorescent proteins potentially explains why GMP-PNP-induced destabilization varies between the two fusion proteins.

Similar trends emerged with GTPγS, albeit with generally minor dimerization effects compared with GMP-PNP. In mGL-Toc34WT, cross-linking indicated 42.6% ± 5.8% dimers and 20.7% ± 13.6% dimers for mEGFP-Toc34WT (Fig. 4 *F*). Though both mGL and mEGFP fusion proteins show a decrease in dimer population in the presence of nonhydrolyzing nucleotides (Fig. 4 *G*), it is observed more through single-molecule studies for mEGFP’s dimer population compared with mGL and vice versa with cross-linking (Fig. 4 *H*). Together, these data reinforce that Toc34’s dimeric state is notably less stable under conditions mimicking GTP binding, consistent with our earlier conclusion that the GDP-bound state (e.g., PDB: 1h65) is the most stable configuration.

### Transit peptide binding strongly destabilizes Toc34 homodimers

In addition to GTP, Toc34’s other key substrate is the transit peptide (tp), whose binding modulates the GTPase cycle. Building on our prior results, which demonstrated that GTP-like conditions favor a more “open” or monomeric state, we next examined how the SS-tp affects Toc34 dimerization. Toc34 recognition in vitro is driven primarily by sequence-independent physicochemical features rather than primary sequence specificity. In our prior work, reversed transit peptides (native vs. reversed sequence orientation, preserving composition) were still competent for Toc34-dependent recognition in vitro, consistent with sequence-independent binding determinants (47). Thus, without a structural or mechanistic recognition rule for Toc34, many candidate disordered peptides (including length-matched random coils) could still exert measurable effects. So, to observe a relative effect from a control, we included interaction with a well-characterized and widely used disordered protein (RCMLa), which is not targeted to the chloroplast, to test whether this effect is specific to SS-tp.

**Figure 5:**
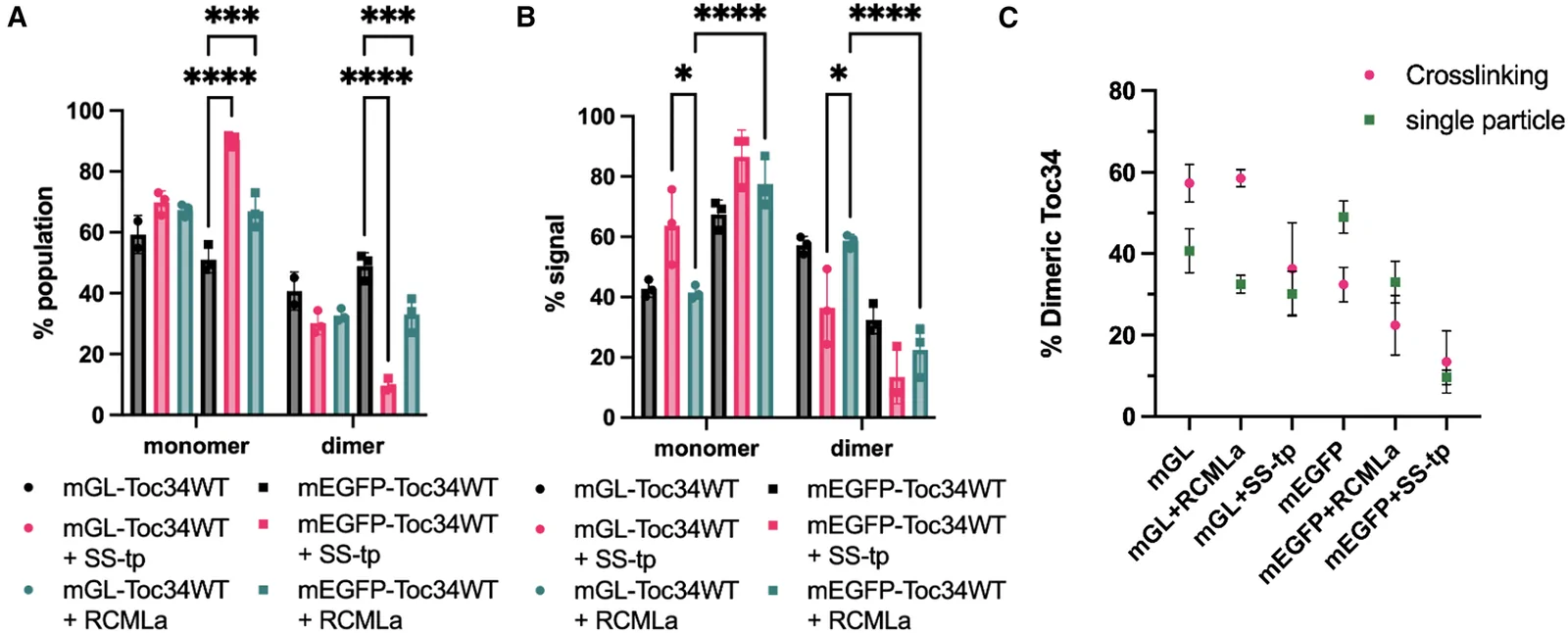
Influence of peptides on Toc34 fusion protein. (*A*) Bar plots made with quantified single-molecule data of mGL-Toc34WT and mEGFP-Toc34WT in the presence of 150 μM SS-tp (small subunit RuBisCO transit peptide, 11.2 kDa molecular weight) in pink and 150 μM RCMLa (an unstructured protein of 14 kDa molecular weight) in green compared with that of the control in dark gray. (*B*) Bar plots made by quantifying immunoblots (Fig. 4*G*) of cross-linked mGL and mEGFP fusion proteins in the presence of 150 μM SS-tp and 150 μM RCMLa. (*C*) Scatter plot comparing the dimer population obtained by cross-linking and single-particle analysis. Bar plots are made with data quantified from replicate experiments, and the error bars represent 95% confidence intervals generated in GraphPad Prism. *p*-values are from two-way ANOVA analysis. ^∗^, *p* ≤ 0.05; ^∗∗^, *p* ≤ 0.01; ^∗∗∗^, *p* ≤ 0.001; ^∗∗∗∗^, *p* ≤ 0.0001.

Using both single-molecule and cross-linking assays, we observed that SS-tp binding consistently reduces the fraction of Toc34 dimers. Through our single-molecule studies, in the presence of SS-tp, mEGFP-Toc34WT displayed 9.6% ± 1.8% dimers and 33.0% ± 5.1% dimers in the presence of RCMLa (Fig. 5 *A*). Similarly, mEGFP-Toc34WT showed 13.4% ± 7.6% dimers in the presence of SS-tp and 22.5% ± 7.3% dimers in the presence of RCMLa by cross-linking (Fig. 5 *B*). These findings align with our earlier observations that the mEGFP tag can sometimes yield more pronounced effects in single-molecule assays (e.g., Fig. 4 *H*). In contrast, mGL-Toc34 often shows more dramatic changes via cross-linking, detecting high levels of dimers. We observed 36.3% ± 11.3% dimers in the presence of SS-tp and 58.5% ± 2.1% dimers in the presence of RCMLa via cross-linking (Fig. 5 *B*), and we observed 30.1% ± 5.5% dimers from mGL-Toc34WT in the presence of RuBisCO small subunit transit peptide (SS-tp) and 32.5% ± 2.2% dimers in the presence of RCMLa via a single molecule (Fig. 5 *A*). This aligns with our previous findings that mGL constructs are more flexible and form excessive dimers compared with mEGFP constructs.

Despite these methodological nuances, both techniques—the ensemble chemical cross-linking data and the single-molecule photobleaching—agree with each other and reflect how transit peptide binding destabilizes Toc34’s dimeric interface, pushing the equilibrium toward monomers. Although nonspecific interactions or molecular crowding may slightly shift the oligomeric balance, SS-tp exerts a substantially more potent and targeted influence on Toc34 conformation (Fig. 5 *C*). Taken together with our earlier findings that nonhydrolyzing GTP analogs also shift Toc34 toward monomers, these data suggest that transit peptide binding, much like GTP binding, favors an “open” conformation of Toc34.

**Figure 6:**
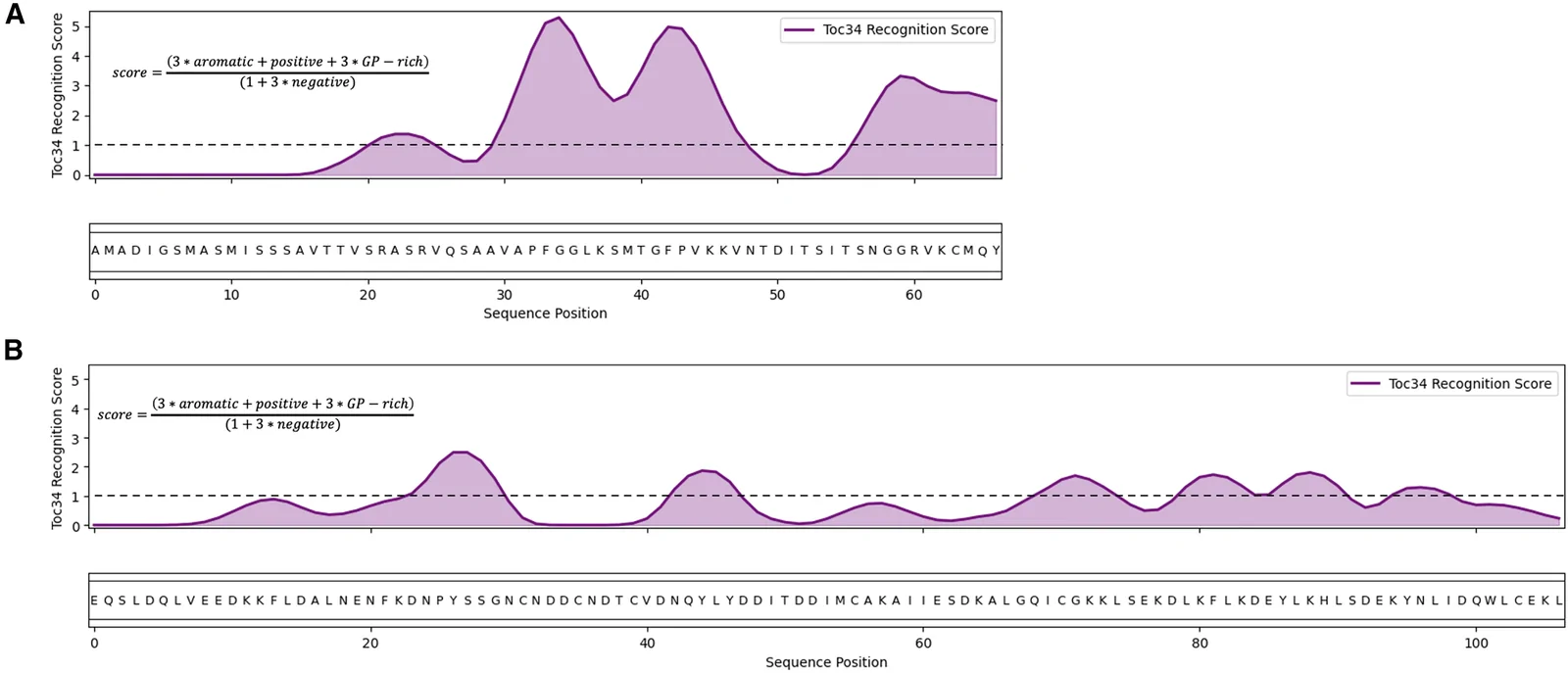
Scoring SS-tp and RCMLa sequences to compare their propensity toward Toc34. (*A*) Toc34 recognition score plotted against the SS-tp sequence. (*B*) Toc34 recognition score plotted against RCMLa sequence. The black dotted line acts as a pointer to compare the scores between the two plots.

To investigate sequence-based origins of this behavior, we compared SS-tp and RCMLa for primary features known to promote Toc34 recognition. Transit peptides are enriched in positively charged, aromatic, and glycine/proline-rich regions and contain relatively few acidic residues (29,48). We quantified these features to generate a Toc34-recognition score and plotted the scoring profile for each sequence (Figs. 6, *A* and *B*). RCMLa is considerably more acidic than SS-tp (Fig. S8) and therefore differs substantially from canonical transit peptides; accordingly, its cumulative feature score reflects a relatively low predicted recognition by Toc34.

Nevertheless, the small but detectable scores across RCMLa suggest that weak recognition is still possible—particularly when the peptide is supplied in molar excess. Though transit peptides play a key role in protein import, noncanonical peptides have also been shown to enter chloroplasts based on their sequence properties (49), and transit peptides themselves are highly diverse (47), underscoring the flexibility of the chloroplast import machinery in engaging a broad range of peptide features. This explains why the Toc34 homodimer remains sensitive to RCMLa despite its non-TP-like characteristics. Additionally, all measurements were performed using equal molar concentrations (not equal mass). Because RCMLa is larger than SS-tp (∼14 kDa vs. ∼11.2 kDa), an equimolar RCMLa condition introduces ∼1.25-fold more residue content into the reaction mixture, which can plausibly enhance weak, nonspecific interactions. This provides a simple explanation for the intermediate effect of RCMLa, whereas SS-tp produces a stronger and biologically relevant stabilization effect.

### smFRET analysis reveals conformational dynamics of Toc34 in its GDP-bound form

**Figure 7:**
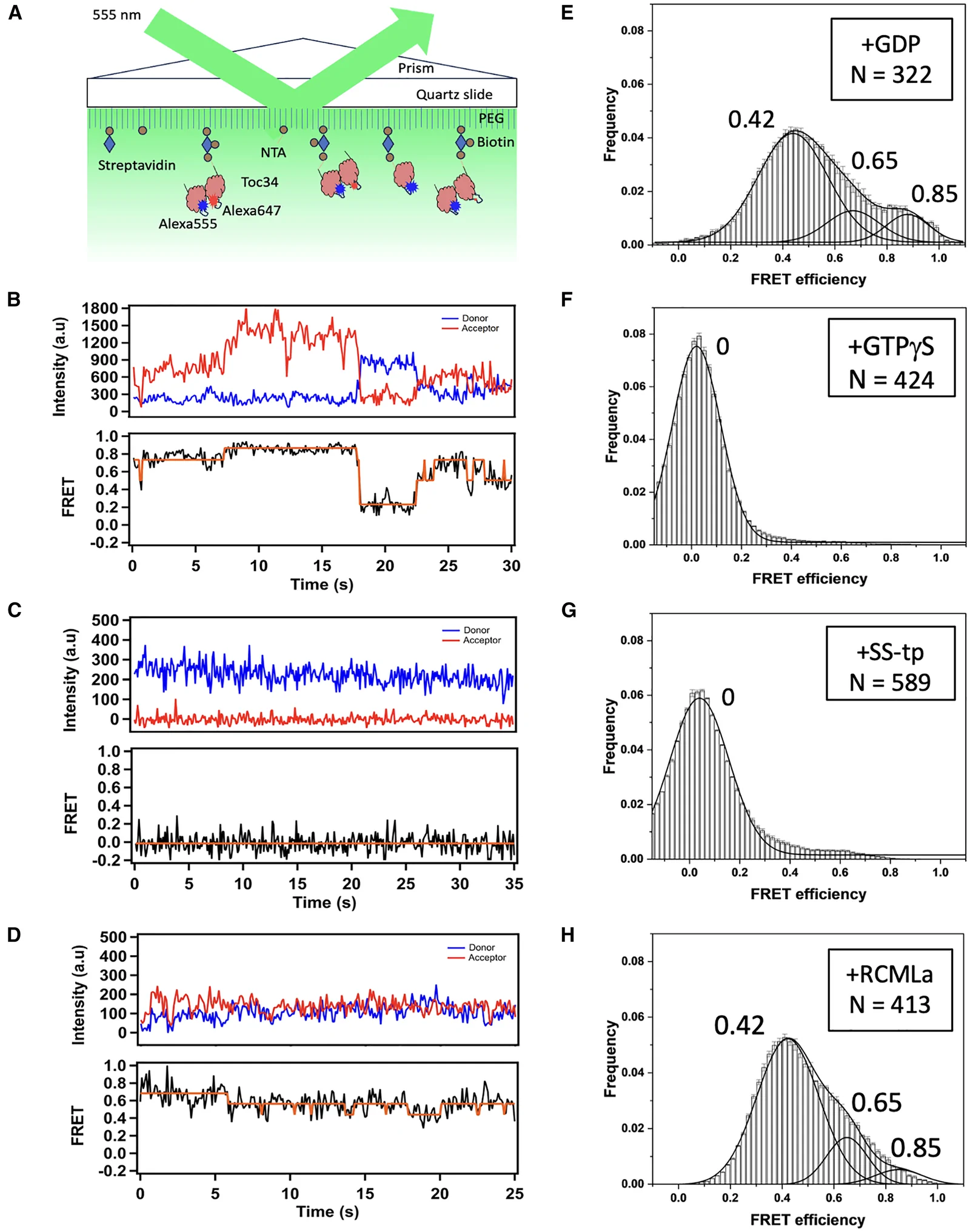
Schematic of smFRET experiments of Toc34 in the presence of its substrates. (*A*) Illustration of prism TIRF with the Alexa 555 labeled Toc34WT as the donor and Alexa 647 labeled Toc34WT as the acceptor, immobilized on the quartz slide with biotinylated NTA bound to streptavidin. (*B*) A representative single-molecule fluorescence intensity time trace of the Toc34WT sample in the presence of GDP. The top panel shows the donor in blue and the acceptor in red, and the bottom panel shows the corresponding FRET trace in black, with the HMM fit in orange. (*C*) is in the presence of GTPγS or SS-tp. (*D*) is in the presence of an unstructured peptide, RCMLa. (*E*) Histogram of all traces combined with Gaussian fitting for the Toc34WT sample in the presence of GDP. (*F*) is in the presence of GTPγS, (*G*) is in the presence of SS-tp, and (*H*) is in the presence of RCMLa.

Toc34 homodimers exist in multiple conformational states in their GDP-bound form, suggesting a dynamic structural landscape that may play a regulatory role in chloroplast protein import. Although the GTPase domain of Toc34 includes a well-characterized arginine finger that stabilizes dimerization through intermolecular interaction, the contribution of a conserved cysteine residue located within a flexible, unstructured loop remains poorly understood. To investigate the conformational dynamics of the Toc34 homodimer and assess the role of this conserved cysteine, we performed smFRET experiments using site-specific fluorescent labeling. Toc34 monomers containing the conserved cysteine were labeled with either Alexa Fluor 555 (donor) or Alexa Fluor 647 (acceptor). The donor-labeled Toc34 was immobilized on a quartz surface, and the acceptor-labeled Toc34 was subsequently introduced into the imaging chamber to enable real-time observation of dimer formation and structural transitions (Fig. 7 *A*). The proximity between fluorophores was monitored through FRET efficiency, which reports on the distance between the labeled residues within the dimer interface.

In the GDP-bound state, Toc34 homodimers exhibited a high FRET signal, consistent with close spatial proximity between donor and acceptor fluorophores (Fig. 7 *B*). In contrast, when either nonhydrolyzable GTP analogs (e.g., GTPγS) or the SS-tp was introduced, FRET efficiency dropped to minimal or undetectable levels (Fig. 7 *C*), indicating a disruption of the dimeric interface and increased separation between the labeled sites. We observe a distinct population showing FRET in the presence of RCMLa, indicating homodimers that are not disrupted by the unstructured peptide (Fig. 7 *D*). Detailed smFRET analysis of the GDP-bound homodimer revealed three distinct FRET efficiency populations centered at 0.42, 0.65, and 0.85 (Fig. 7 *E*). These discrete states likely represent a set of preferred conformational substates, suggesting that the Toc34 dimer samples a range of structural arrangements even within a single nucleotide-bound condition. Given that conformational switching is a hallmark of regulatory GTPases, the presence of these distinct FRET states highlights a potential mechanism by which Toc34 modulates preprotein import through dynamic dimer rearrangements.

Based on the known Förster radius (R_0_) of 51.4 Å for the Alexa Fluor 555/647 dye pair (Fig. S9 *A*), we estimated the distances corresponding to each FRET efficiency state using the inverse sixth-power relationship between energy transfer and distance. These calculations yielded interfluorophore distances of approximately 54.2 Å, 46.4 Å, and 38.5 Å for the 0.42, 0.65, and 0.85 FRET states, respectively (Fig. S9, *B* and *C*). Upon the addition of either GTPγS or SS-tp, these FRET populations shifted markedly toward lower efficiencies or a complete absence of FRET (Fig 7 *F* and *G*), consistent with increased intersubunit separation and disruption of the homodimer interface. These findings corroborate our single-color photobleaching experiments using Toc34 fusion proteins, which also showed that GTP binding or interaction with the transit peptide disrupts dimerization. Similarly, we observe the three FRET states (0.42, 0.65, and 0.85) in the presence of RCMLa (Fig. 7 *H*) among the population holding the homodimer interface. Here, we observe a shift in the FRET peaks toward a lower FRET state (0.42), demonstrating that the peptide RCMLa modulates the conformation of homodimers. Together, our results demonstrate that Toc34 dimerization is sensitive to both nucleotide state and preprotein binding, and that the conserved cysteine serves as a strategically positioned probe to monitor these structural transitions. This study reveals a dynamic, multistate model of Toc34 homodimerization that underpins its function as a conformational switch during chloroplast protein import.

**Figure 8:**
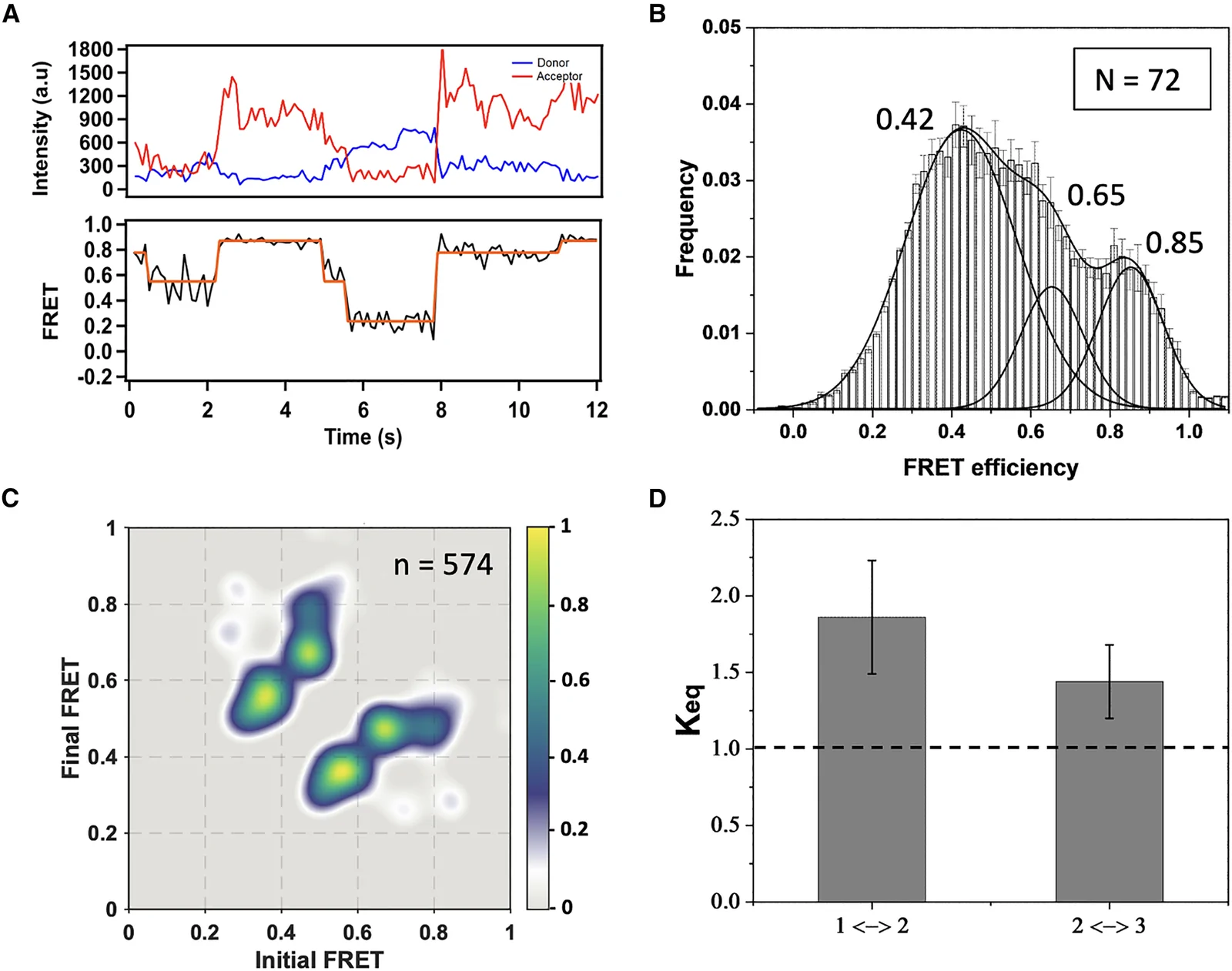
Dynamics of Toc34 dimer observed in its GDP-bound state. (*A*) A representative dynamic single-molecule fluorescence intensity time trace. The top panel shows the donor in blue and acceptor in red, and the bottom panel shows the corresponding FRET trace in black with its HMM fit in orange. (*B*) Histogram of all dynamic traces with Gaussian fitting. (*C*) A transition density plot (TDP) showing a heat map of all transitions observed among the dynamic traces. The “n” corresponds to the total number of transitions observed, and (*D*) is a bar plot showing equilibrium constant for the transitions between states 1 and 2 (0.42, 0.65) and between 2 and 3 (0.65, 0.85). The black horizontal dotted line indicates values of the rate constants equal to 1. Error bars represent 95% confidence intervals generated in Origin Pro.

Among the donor and acceptor trajectories observed in the GDP-bound Toc34 smFRET data set, we identified three distinct classes of FRET traces: dynamic, static, and transient binding events. The dynamic traces (Fig. 8 *A*) exhibit reversible transitions between three well-defined FRET states centered at 0.42, 0.65, and 0.85 (Fig. 8 *B*), consistent with conformational rearrangements within the homodimer, particularly involving the flexible, unstructured loop region. These transitions reflect real-time fluctuations in the proximity of the fluorophores, indicating a high degree of structural plasticity within the dimer interface. This observation is supported by the TDP (Fig. 8 *C*), which maps the frequency and directionality of FRET state transitions across the population.

To quantitatively assess the kinetics of these conformational transitions, we performed dwell time analysis of the dynamic traces. Histograms of dwell times for each pairwise FRET transition were fit to single-exponential decay functions, from which transition rates were extracted (Fig. S10). We found that transitions between FRET states, 0.42 to 0.65, occur more rapidly (1.67 ± 0.26 s^−1^) compared with transitions from 0.65 to 0.42 (0.90 ± 0.11 s^−1^). Similarly, the transitions between 0.65 and 0.85 are faster (0.98 ± 0.13 s^−1^) than that of 0.85 to 0.65 (0.68 ± 0.07 s^−1^). Mean occupancies at each FRET state show more time (2.9 ± 0.7 s) spent at state 1 (0.42) compared with that of state 2 (0.65), with 1.6 ± 0.2 s and 3 (0.85) with 2.2 ± 0.3 s (Fig. S11). We then used these rate constant values to calculate the equilibrium rate constant for transitions between state 1,2 (*k*_*eq*_(1,2)) and 2,3 (*k*_*eq*_(2,3)), as described in the materials and methods section. Both *k*_*eq*_(1,2) and *k*_*eq*_(2,3) values are higher than 1, indicating the equilibrium shift toward a higher FRET state (Fig. 8 *D*). Although transitions from low to high FRET occur more rapidly than the reverse, the population analysis shows that the lower FRET state is the most populated at equilibrium. This suggests that, despite the kinetic bias toward loop compaction, the energetically most favorable conformation corresponds to the extended loop (low FRET). The higher FRET states likely represent transiently compact conformations that are visited frequently but occupied for shorter durations.

In contrast, the static traces (Fig. S12 *A*), which represent the majority (77%) of the GDP-bound population (Fig. S12 *B*), display states locked in one of the three FRET states, indicating reduced conformational mobility and potentially more stable dimeric contacts. A third class of traces consists of transient binding events in which acceptor fluorescence appears only after donor excitation begins—reflecting delayed dimer formation or exchange dynamics (Fig. S13 *A*). Although less frequent under our experimental conditions (8-nM acceptor protein), these transient events occupy the same FRET states (0.42, 0.65, 0.85) as the stable population (Fig. S13 *B*). Most transient traces remain static after acceptor appearance, suggesting formation of a stable complex at a particular FRET level.

Although these results confirm multiple conformations of the Toc34 homodimer in its GDP-bound state, the addition of RCMLa shifts the equilibrium toward the monomeric population. We also observe an increase in the fraction of static homodimer traces (93%), which become more confined to the lowest FRET state (0.42) (Fig. S14). Among the remaining dynamic traces (7%) in the presence of RCMLa, fluctuations are primarily restricted to the 0.42–0.65 states (Fig. S15). These observations align with our single-molecule photobleaching analysis, which revealed a partial shift toward monomers and support the notion that RCMLa perturbs the homodimer interface. Together, the smFRET data reveal an overall shift of the equilibrium toward lower-FRET conformations when RCMLa is present. Importantly, these FRET states observed in the presence of RCMLa likely represent an intermediate conformation of the homodimer, offering insight into how weakly recognized peptides may modulate the early steps of the Toc34-mediated import pathway.

**Figure 9:**
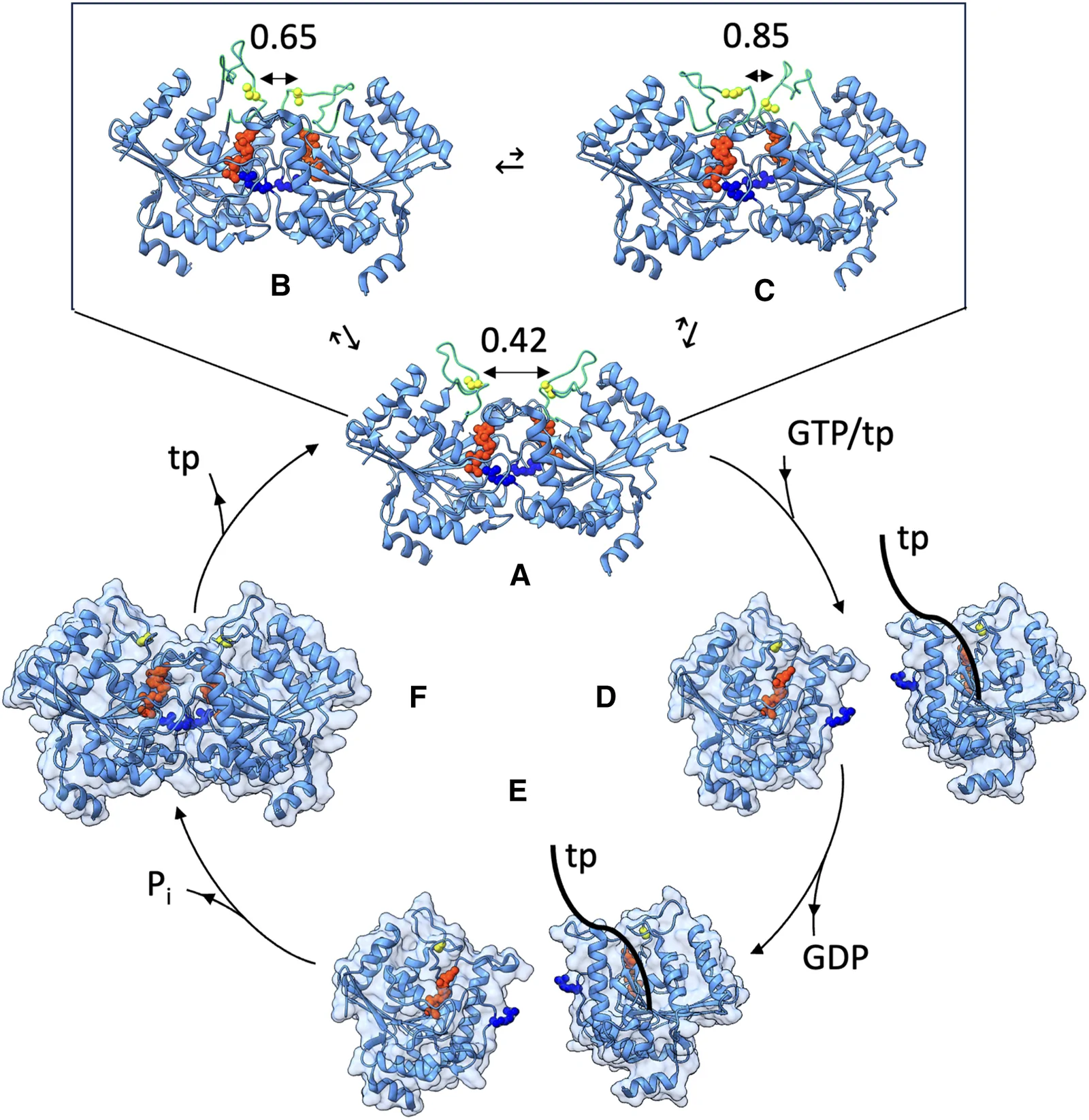
A model illustrating the Toc34 GTPase cycle involved in protein import. (*A*) Toc34 homodimer bound to GDP in its GTPase cycle at a FRET state of 0.42. Toc34 is shown in a cartoon representation in light blue. The conserved cystine (C215) is shown in a yellow ball and stick representation, the flexible loop is highlighted in green, R133 is shown in a dark blue ball and stick representation, the nucleotide is shown in an orange ball and stick representation, and the transit peptide is shown in black. (*B*) is at a FRET state of 0.65 and equilibrium toward 0.42 FRET state. (*C*) is at a FRET state of 0.85 and equilibrium toward 0.65 and 0.42 FRET states. (*D*) With Toc34 bound to GTP and transit peptide, the presence of tp/GTP changes Toc34 conformations, leading to disruption of the homodimer interface. (*E*) With a GTP-bound state, Toc34 enters an intermediate state leading to GTP hydrolysis. (*F*) With the energy released by GTP hydrolysis, the transit peptide is moved toward the neighboring Toc subunit, and Toc34 gets to its stable GDP-bound state as a homodimer. Toc34 is shown in a cartoon representation in light blue. The conserved cystine (C215) is shown in a yellow ball and stick representation, the flexible loop is highlighted in green, R133 is shown in a dark blue ball and stick representation, the nucleotide is shown in an orange ball and stick representation, and the transit peptide is shown in black.

From our findings, we updated the mechanistic model of protein import, in which Toc34 acts as a nucleotide- and substrate-sensitive conformational switch. In its GDP-bound form, Toc34 populates three FRET states, with the equilibrium shifted toward the lowest FRET state (0.42) (Fig. 9 *A*), representing a dimeric architecture stabilized by the arginine finger and potentially additional contacts within the unstructured loop. Under these conditions, transitions between a compact dimer (higher FRET states, 0.65 and 0.85) (Fig. 9, *B* and *C*) and a less compact dimer (0.42) are still observed, indicating that the GDP-bound dimer remains flexible and can sample multiple closed conformations. These conformations might play a crucial role in interacting with an incoming peptide.

We then show that the presence of SS-tp/GTP alters the homodimer conformation (Fig. 9 *D*), disrupting the interface and leading to monomer formation. From our single-molecule photobleaching and smFRET experiments, we observe that Toc34 exists as a stable homodimer until GTPγS or SS-tp is introduced, making the homodimer an autoregulatory “standby” state that tunes receptor sensitivity. This state is likely to precede the hydrolysis state that leads to a stable GDP-bound dimer (Fig. 9 *F*). Between hydrolysis and homodimer formation, we expect a transit peptide handoff to other subunits (Fig. 9 *E*), which might explain heterodimer formation between Toc34 and Toc159, facilitating the transfer of transit peptide to Toc75, the pore, and then to TIC. Toc34–Toc159(120) heterodimers represent the commitment/gating state that couples preprotein recognition to GTPase cycling and handoff to TIC. Consistent with a competitive balance between homo- and heterodimer states, our preliminary association/competition experiments indicate that weakening the Toc34 homodimer interface may increase Toc34–Toc159 association (data not shown), supporting a model in which reduced homodimer stability facilitates the transition toward an import-committed heterodimeric receptor state. These heterodimer interactions are currently being investigated, and we plan to expand our studies in the future. Our smFRET experiments so far reveal the dynamics and kinetic equilibrium of the GDP-bound state conformations of Toc34 homodimer, demonstrating their flexibility.

In contrast, the presence of RCMLa restricts this dynamic landscape, confining the homodimer predominantly to lower FRET states (0.42 and 0.65). This behavior is consistent with RCMLa stabilizing a partially closed intermediate—potentially corresponding to the A → D transition in Fig. 9—between the more flexible GDP-bound dimer and the monomeric state. Conversely, the addition of GTP-like analogs or the SS-tp promotes dimer dissociation or shifts the receptor toward a more open, flexible conformation. Notably, SS-tp alone is sufficient to induce large conformational changes, even in the absence of GTP, highlighting its function as a specific ligand that directly engages the Toc34 dimer interface. This also suggests that peptide recognition alone can be energy independent.

Together, these results support a model in which both nucleotide cycling and precursor binding regulate the oligomeric state of Toc34. Such multistate regulation provides multiple checkpoints for tuning receptor dimerization, substrate recognition, and precursor handoff to the TOC translocon, ultimately enhancing the specificity and efficiency of chloroplast protein import.

## Discussion

The paper investigates the dynamics of Toc34 oligomerization, a key component of the TOC, which is crucial for posttranslational protein translocation into chloroplasts. Toc34, a GTPase, plays a regulatory role through its ability to form homo- and hetero-oligomers. This study employed single-molecule photobleaching and cross-linking assays to investigate how substrate-mediated dynamics affect Toc34’s conformational states.

The study employed fluorescent proteins, mGL and mEGFP, fused to Toc34, to monitor oligomeric states. Although both tags provided valuable data, they exhibited different photophysical properties that influenced the results. mGL showed better stability in SDS-PAGE, aiding in-gel detection of dimers, but displayed slower, time-dependent photobleaching in single-molecule TIRF experiments, requiring a specific intensity-subtraction method for analysis. In contrast, mEGFP had discrete photobleaching steps but lost fluorescence during SDS-PAGE. The mutated residues in mGL resulted in a more flexible structure, which may have contributed to its increased stability and resistance to heat compared with mEGFP. (26). Both tags, however, corroborated the findings that the GDP-bound state of Toc34 favors a stable dimer, whereas GTP-like conditions and transit peptide binding destabilize this dimer, promoting monomeric or open conformations. The importance of the arginine finger motif (Arg133) for stable homodimer formation was confirmed through the R133A mutant, which showed significantly reduced dimer populations across both mGL and mEGFP fusions. R133A mutants are known to lack the ability to hydrolyze GTP (18). This highlights the structural role of this motif in maintaining dimeric assembly, particularly in the GDP-bound state, and its associated function.

In parallel to single-molecule measurements, cross-linking provided an orthogonal method to quantify Toc34’s oligomeric state. mGL’s fluorescence stability was particularly beneficial here, enabling the rapid detection of cross-linked dimers without secondary immunodetection (50,51). Cross-linking data converged with single-molecule results, providing high confidence that variations in dimer fractions reflected actual oligomeric changes rather than artifacts of labeling or detection. This convergence highlights the importance of combining distinct methodologies—a strategy employed in previous investigations of chloroplast protein import (44,52,53), including seminal work from the Bruce laboratory (29,54).

The research found that the dimeric state of Toc34 is disrupted by the presence of the SS-tp and nonhydrolyzable nucleotides, such as GMP-PNP and GTPγS. These findings suggest that substrate interactions and the oligomeric state of TOC subunits are critical in regulating protein import. Specifically, nonhydrolyzing nucleotides, which mimic the GTP-bound state, destabilize the Toc34 homodimer, promoting a more “open” conformation. Likewise, SS-tp binding significantly reduced dimer levels, supporting a model in which transit peptides also promote an open Toc34 conformation, facilitating precursor protein recognition and import. This finding complements earlier work that implicates Toc34 in the engagement of transit peptides during the initial stages of translocation (10,11,21,29). Notably, when we used a nonspecific disordered protein (RCMLa) as a control, it exerted an intermediate effect on dimer stability, indicating the relative influence of peptides and the specificity of SS-tp on Toc34 dimerization. Analysis of the RCMLa sequence revealed low levels of Toc34-recognition features, reinforcing that Toc34 engages substrates through a low-specificity recognition mechanism.

The paper highlights that Toc34 functions as a gating mechanism through its homodimer dynamics, regulating protein import. The conformational switching between a GDP-bound dimeric state and GTP- or transit peptide-bound monomeric states is proposed to be crucial for gating precursor protein entry into the chloroplast. This regulated dimerization is a pivotal factor in chloroplast protein import, with both nucleotides and transit peptides reshaping the oligomeric landscape of Toc34. Our smFRET experiments specifically highlight the Toc34 homodimer dynamics by directly targeting the conserved C215 residue for labeling.

We observed three distinct FRET efficiency states—0.42, 0.65, and 0.85—in the GDP-bound form of Toc34, indicating the presence of multiple, interconvertible conformations within the stable homodimer. These conformational states likely arise from the flexibility of a long surface-exposed loop containing a conserved cysteine residue, which served as the site for site-specific fluorophore labeling in our smFRET experiments. Variations in FRET efficiency reflect dynamic changes in the distance between donor and acceptor fluorophores and thus report directly on the structural mobility of this loop region. Dwell time analysis revealed that transitions from lower to higher FRET states occur faster than the higher to lower FRET states. The equilibrium constants *k*_*eq*_(1,2) and *k*_*eq*_(2,3) were 1.86 ± 0.37 and 1.44 ± 0.24, respectively, both favoring movement toward higher FRET states, with a slight preference for state 2 (0.65). These results are consistent with a greater structural rearrangement and a more stable lower-FRET conformation. Overall, the smFRET data indicate conformational switching on the timescale of several hundred milliseconds to a few seconds.

Although the precise in vivo dynamics of Toc34 oligomerization and dissociation remain unknown, available kinetic data on preprotein import into chloroplasts provide important contextual insights. Radiochemical binding assays have estimated the number of preprotein binding sites per chloroplast to be approximately 1800 (12,54). In addition, in vitro import experiments using the precursor to the small subunit of RuBisCO (prSSU) in pea chloroplasts report maximum import rates of ∼8000 counts per minute (cpm) (12). Based on these values, the average time required for importing a single preprotein, assuming one preprotein per binding site, is approximately 75 ms. Additionally, single-molecule electrophysiological studies of the protein import-related anion channel, which is thought to reflect preprotein movement through TIC/TOC translocons, report a prSSU-induced channel closure time of approximately 989 ± 214 ms (12). Together, these findings suggest that the dynamics of protein translocation through the TOC complex occur on a timescale ranging from ∼75 ms to ∼1000 ms. This range closely corresponds to the smFRET-derived Toc34 conformational transition rates we observed, occurring on the order of 680 to 2000 milliseconds, indicating that the nucleotide- and substrate-dependent dimer dynamics of Toc34 are well matched to the physiological timescale of chloroplast preprotein import. Additionally, the GTP hydrolysis rate of psToc34 (*k*_*cat*_ = 0.037 ± 0.001 min^−1^) suggests a slow intrinsic enzymatic turnover (18), indicating that dimer-monomer transitions may be the rate-limiting regulatory step in import. Taken together, these data indicate that the conformational transitions within the Toc34 homodimer—particularly those governed by loop dynamics—are well matched to the functional timescale of protein translocation in vivo. Thus, the smFRET-detected fluctuations likely correspond to key regulatory motions involved in substrate binding or signal transduction within the TOC complex.

By contrast, in the presence of nonhydrolyzable GTP analogs (e.g., GTPγS) or the transit peptide (SS-tp), the FRET states shift toward lower efficiencies or are abolished entirely, indicating an increase in interfluorophore distance and a disruption of the homodimer interface. These observations support a model in which GTP binding or interaction with the transit peptide promotes a more open, flexible, or monomeric conformation of Toc34. Interestingly, RCMLa resulted in a more restricted conformational state of Toc34 homodimers, spanning 0.42–0.65 FRET states, suggesting that a peptide-bound homodimer would have a loosely arranged interface. Collectively, these results reveal that Toc34 exists in a dynamic equilibrium of structural states modulated by nucleotide and substrate binding. GDP stabilizes a compact, dimeric form, whereas GTP analogs and transit peptides shift the equilibrium toward more dynamic or dissociated states. These conformational transitions likely underlie a gating mechanism that controls the initiation and progression of chloroplast protein import.

Our smFRET results demonstrate that Toc34 exists in multiple interconverting conformational states in its GDP-bound form, with transitions occurring on a subsecond timescale that is physiologically relevant to chloroplast protein import. These dynamics are likely mediated, at least in part, by the flexibility of an unstructured surface loop containing a conserved cysteine residue. Importantly, recent high-resolution cryo-EM structures (9,43) of the TOC complex (e.g., PDB: 7XZI and 7VCF) have resolved a short segment of the Toc34 transmembrane (TM) domain—specifically spanning residues F326 to D397—which corresponds to the C-terminal region removed in our psToc34 ΔTM268 truncation construct used for smFRET labeling. This C-terminal segment, rich in glycine and proline residues, may contribute to the proper positioning and dynamic tethering of the GTPase domain relative to the Toc75 translocation pore in the native membrane environment. Its absence in our construct may influence the conformational range or stabilization of specific FRET states, potentially underestimating long-range structural communication between the membrane anchor and the GTPase domain. Future studies using full-length, membrane-reconstituted Toc34 will be essential to evaluate the role of this region in modulating dimerization dynamics and interaction with other TOC components.

Furthermore, both cryo-EM structures reveal that the N-terminal GTPase domains of Toc34 and Toc120 are unresolved, despite their presence and confirmation by mass spectrometry (9,43). This absence, together with AlphaFold3 (55) predictions of flexible linkers connecting the GTPase and TM domains in both proteins, supports a model in which the cytosolic GTPase domains are highly mobile. Such flexibility may be essential for the conformational rearrangements required during transit peptide recognition, receptor dimerization, and handoff to the Toc75 channel. The flexibility of G-proteins enables homo- and heterodimerization, thereby modulating the state of the TOC complex that governs protein import. Toc34 homodimers function as autoregulatory receptors in a closed conformation. Binding of a transit peptide to the Toc34 homodimer induces a conformational change that disrupts the homodimer interface and permits heterodimerization with Toc159(120). Toc34 homodimer, as a first state in protein import, is sensitive to transit peptide and acts as a low-commitment step with incoming peptides, preventing inappropriate opening. Whereas, the heterodimer is a second state that controls protein import by its association with other subunits. Recent cryo-EM structures (9,43) reveal that the β-barrels of Toc120 and Toc75 adopt a hybrid state, suggesting that association of Toc34 with Toc120 establishes a physical connection to Toc75, the translocation pore, thereby facilitating protein import. The coordinated association of all TOC subunits implies the formation of a molecular bridge that enables the preprotein to traverse the membrane. At the same time, it is important to recognize that *Chlamydomonas* is an algal system that differs substantially from land plants, with distinct TOC/TIC homologs; consequently, the organization of the import machinery in land plants remains ambiguous in the absence of a definitive structure.

In this context, our smFRET analyses reveal previously unresolved conformational dynamics of the Toc34 GTPase domain in solution, helping to close critical gaps in the mechanistic understanding of chloroplast protein import while also carrying several limitations that necessarily temper interpretation. Most notably, we examined only the soluble, cytosolic GTPase domain of Toc34 expressed in *E. coli*, rather than full-length Toc34 embedded in the native outer chloroplast envelope. This construct lacks the TM segment and membrane environment that could influence conformational equilibria, nucleotide exchange, and dimerization kinetics; nevertheless, the soluble domain is the form that has been most extensively characterized biochemically (18,56) and structurally (7), providing a strong and widely comparable reference framework for interpreting our measurements. A second limitation is that our experiments were performed in a reductionist system lacking the full TOC context—particularly Toc75, as well as the other receptor GTPases (e.g., Toc159, Toc120)—which are expected to impose additional structural constraints, alter local effective concentrations and avidity, and potentially reshape the kinetic landscape of Toc34 association and conformational switching. Thus, some aspects of the dynamics we observe may differ in magnitude—or even in mechanism—within the intact import apparatus. Importantly, however, the high spatiotemporal resolution of our assay establishes a quantitative baseline for Toc34 behavior in isolation, creating a foundation for future reconstitution experiments that can directly evaluate how membrane embedding and additional TOC subunits perturb Toc34 dynamics and regulatory transitions.

Though our current work is necessarily in vitro, we plan to extend this single-molecule strategy to membrane-bound systems in future studies. Together, these observations support the idea that regulated conformational plasticity is a defining feature of TOC complex function. Moving forward, we aim to expand this approach to other TOC subunits—particularly Toc120 and Toc159—to build a more complete model of the dynamic and coordinated events that govern chloroplast protein import.

## Data and code availability

Custom MATLAB codes for automated trace analysis and Python code for error calculations and peptide sequence comparison are available on the GitHub repository: https://github.com/sreekavyapenneru/Single-molecule.

## Acknowledgments

Research in the Lamichhane lab is supported by the 10.13039/100000002NIH grant R35GM142946 (R.L.). The authors also acknowledge financial support from the Charles P. Postelle Distinguished Professorship of Biotechnology and the NSF grant IOS-2233695 (B.D.B.).

## Author contributions

S.K.P.: writing – review & editing, writing – original draft, visualization, methodology, software, validation, investigation, formal analysis, and conceptualization. S.T.-K.: writing – review & editing, software, data curation, and validation. R.L.: writing – review & editing, supervision, resources, project administration, funding acquisition, and conceptualization. B.D.B.: writing – review & editing, writing – original draft, supervision, resources, project administration, funding acquisition, and conceptualization.

## Declaration of interests

The authors declare no competing interests.

## Supporting Material

Document S1. Figures S1–S15 and Tables S1 and S2

Document S2. Article plus Supporting Materials
