# Supplementary material for: Single-molecule fluorescence and cross-linking reveal ligand-gated Toc34 oligomerization dynamics
Source: Biophys J. 2026 Feb 10;125(6):1475–93. doi: 10.1016/j.bpj.2026.02.006 (PMC12973003; doi:10.1016/j.bpj.2026.02.006)
Supplement: Document S1. Figures S1–S15 and Tables S1 and S2 [file mmc1.pdf]

Biophysical Journal, Volume *125*

## Supplemental information

# Single-molecule fluorescence and cross-linking reveal ligand-gated Toc34 oligomerization dynamics

**Sree Kavya Penneru, Sriram Tiruvadi-Krishnan, Rajan Lamichhane, and Barry D. Bruce**

**Penneru et al. (2025)**

**Supplemental information:**

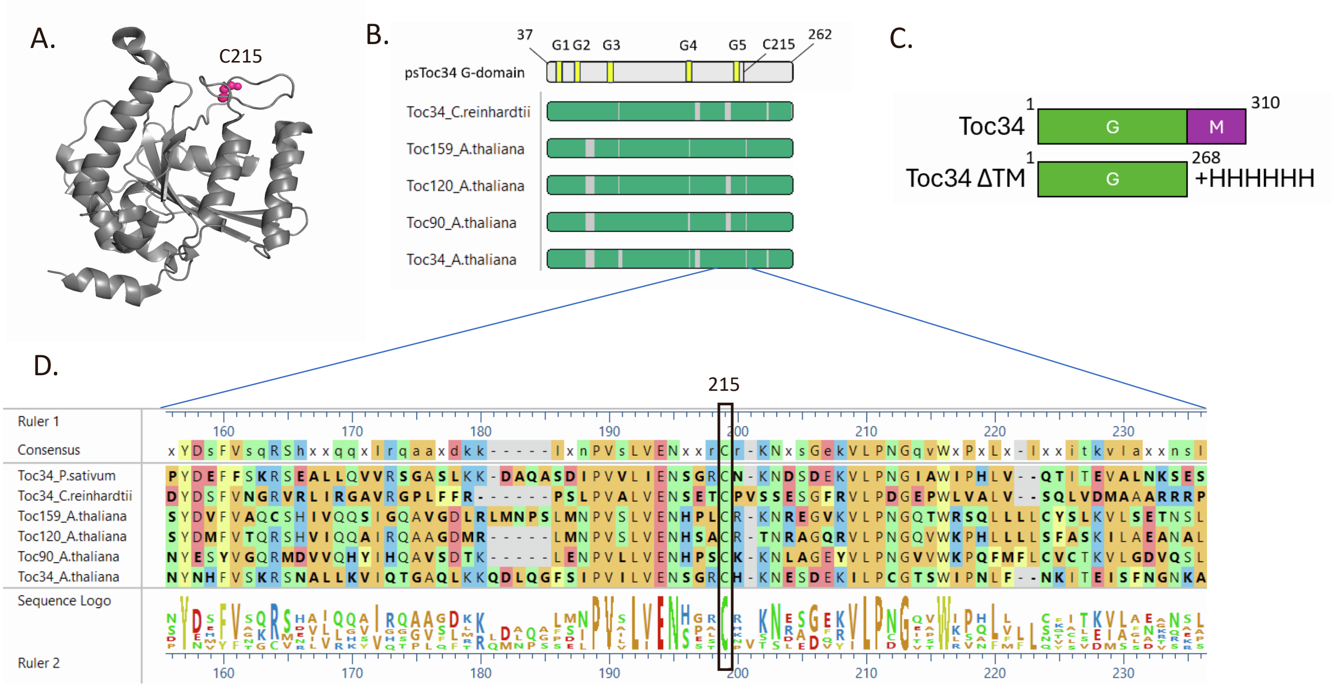


**Figure S1. Toc34 structure and its conserved domains.** (*A*) Crystal structure (1h65) of psToc34 monomer showing C215 in pink space filling. (*B*) a general overview of Toc subunits aligned using Clustal Omega, showcasing domains with high similarity in green. The five sub-domains in the G-domain are shown in yellow. (*C*) Illustration of Toc34 full-length construct with a GTPase (G) domain and a membrane (M) domain, followed by the soluble construct made by truncating the M domain from residue 268 and with a 6x His-tag. (*D*) multiple sequence alignment of different Toc subunits and their homologs via Clustal Omega to show conservation of Cystine at position 215 of psToc34.

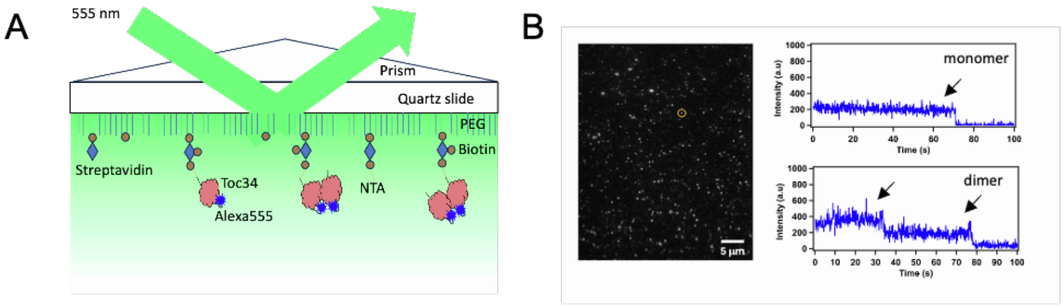


**Figure. S2 Illustration of TIRF microscopy and representative single-molecule fluorescence trajectories.** (*A)* Illustration of prism TIRF with the Alexa 488 labelled Toc34 immobilized on the quartz slide with biotinylated NTA bound to streptavidin. (*B)* Representative TIRF image (Left) of single molecules imaged via TIRF microscope and single-molecule fluorescence intensity time trajectories (Left) of Alexa 488 labelled Toc34 protein. The top panel shows a single-step photobleaching representing a monomer, and the bottom panel shows a double-step photobleaching representing a dimer.

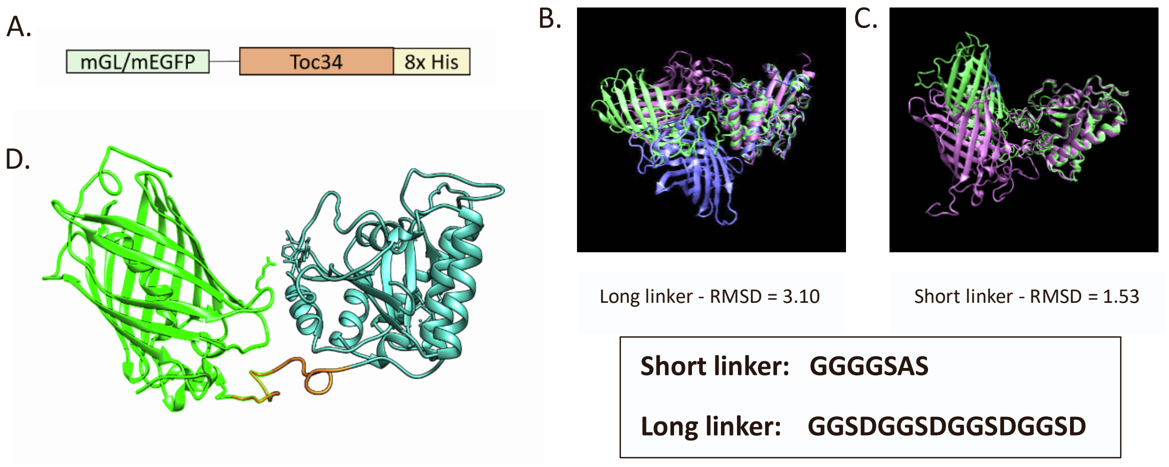


**Figure S3. Design and structure prediction of fusion protein.** *(A)* Fusion protein construct with the fluorophore attached at the N terminus with a short linker sequence and an 8x His-tag attached at the C-terminal. (*B)* Alignment of three homology models of the WT mGL fusion protein with the long linker, and the respective average RMSD of Toc34 and the linker's backbone. (*C)* Alignment of three homology models of the WT mGL fusion protein with the short linker, and the respective RMSD of Toc34 and the linkers backbone. (*D*) A representative structure of a fusion protein showing fluorescent protein in green, short linker in orange, and Toc34 in cyan.

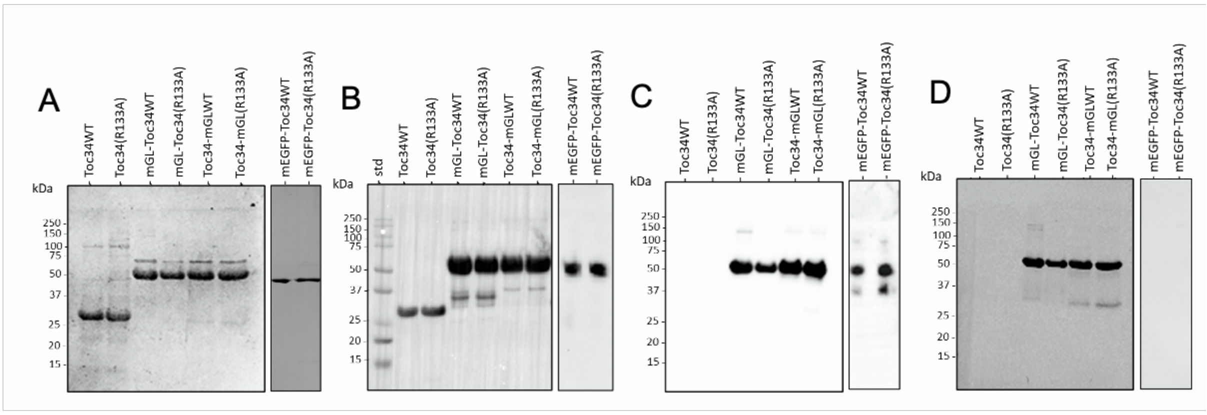


**Figure. S4 Characterization of mGL-Toc34 and mEGFP-Toc34 fusion proteins.** (*A)* Coomassie blue-stained SDS-PAGE of the purified proteins used in the paper: psToc34WT, psToc34(R133A), four mGL-containing fusion proteins, and the two mEGFP C-terminal fusions. (*B)* Immunoblot of the same gel shown in *A* using a polyclonal antibody against psToc34. (C) Immunoblot of the same gel shown in (*A*) using a polyclonal antibody against GFP. (*D)* a stain-free, *in-gel* fluorescence detection of mGL-containing fusion proteins following SDS-PAGE. Note that mEGFP loses its chromophore during SDS-PAGE.

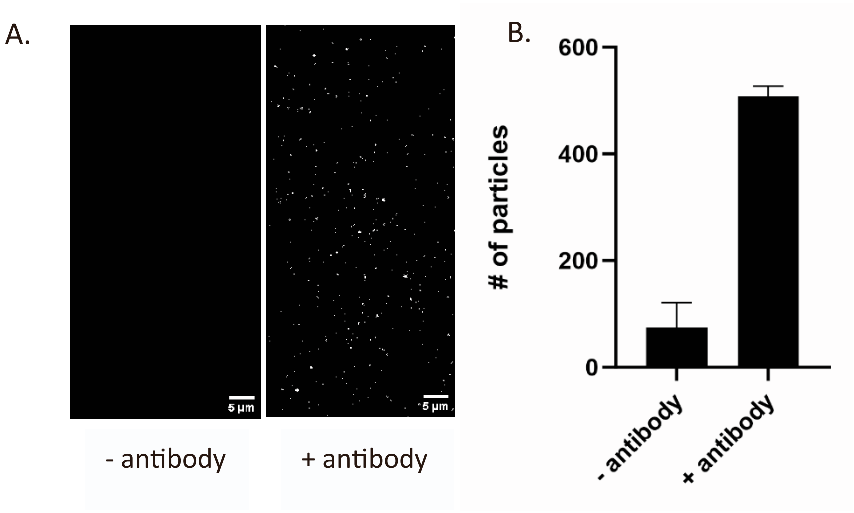


**Figure S5. Negative control for immobilization of protein.** *(A),* A representative image captured by the TIRF microscope with no antibody in the left panel and with an antibody in the right panel. (*B)* A bar plot obtained by quantifying the number of particles captured by the TIRF image using our MATLAB code.

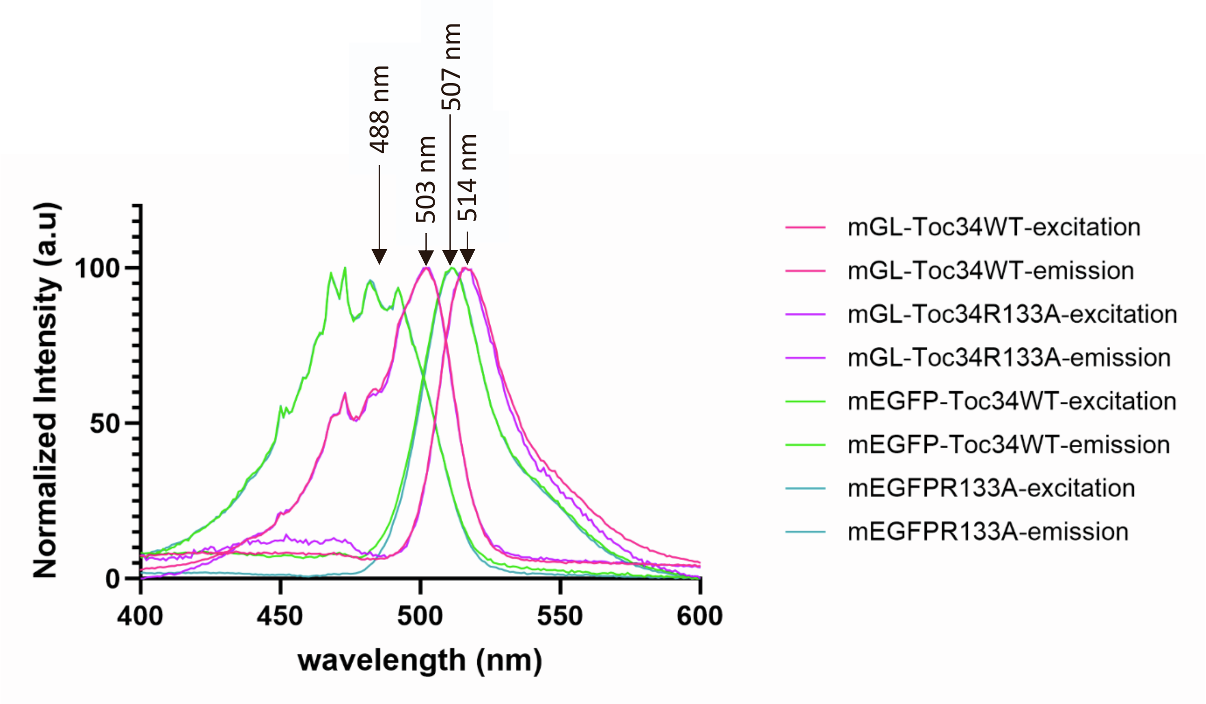


**Figure S6. Excitation and emission spectra of fusion proteins.** mGL-Toc34 fusion proteins excite at 503 nm and emit at 514 nm, whereas mEGFP-Toc34 fusion proteins excite at 488 nm and emit at 510 nm, as expected by their respective fluorophores. All readings have been averaged over three replicates.

| Feature | mGL | mEGFP |
|---|---|---|
| Molecular weight (kDa) | 26.8 | 27 |
| Excitation $\lambda$ (nm) | 503 | 488 |
| Emission $\lambda$ (nm) | 514 | 507 |
| Half-life $t_{1/2}$ (s) | 30.8 at 70 $\mu$W laser power | 239.0 at 60 $\mu$W laser power |
| Brightness | 73.3 | 33.6 |
| Quantum yield | 0.72 | 0.6 |
| Maturation time (min) | 13.5 | 14.5 |
| Maturation efficiency (%) | 90 | 70 |

**Table S1. Photochemical and spectral properties of mGL and mEGFP fluorescent proteins.**

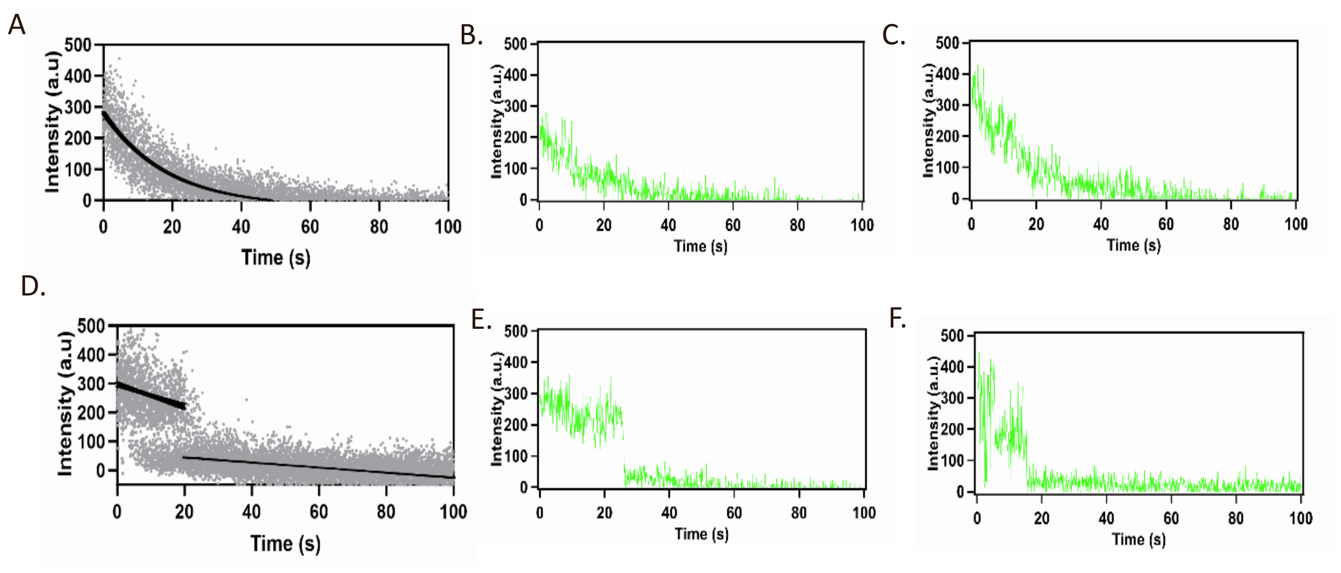


**Figure S7. Representative single-molecule traces of mGL and mEGFP fusion proteins.** (*A)* Non-linear one-step decay fit over 20 traces of mGL fusion protein. The thickness of the fitted line corresponds to the error in fitting. (*B)* A representative trace for Toc34 monomer from mGL fusion protein. (*C)* A representative trace for Toc34 dimer from mGL fusion protein. (*D)* Two separate linear fits over 20 traces of the mEGFP fusion protein, before and after photobleaching. The thickness of the fitted line corresponds to the error in fitting. (*E)* A representative trace for Toc34 monomer from mEGFP fusion protein. (*F)* a representative trace for Toc34 dimer from mEGFP fusion protein.

| Sample condition | Single molecule (sm) | | | Crosslinking (cx) | | Δ |sm-cx| |
|---|---|---|---|---|---|---|
| | M % | D % | n | M % | D % | |
| Toc34WT | - | - | - | 56.3±3.3 | 43.7±3.3 | - |
| Toc34(R133A) | - | - | - | 85.3±8.2 | 14.7±8.2 | - |
| mGL-Toc34WT | 59.3± 5.4 | 40.7±5.4 | 1551 | 42.7±4.6 | 57.3±4.6 | 16.6 |
| mGL-Toc34(R133A) | 83.7±3.0 | 16.3±3.0 | 1437 | 86.7±6.0 | 13.3±6.0 | 7.0 |
| mGL-Toc34WT + GTP | 58.0±3.0 | 42.0±3.0 | 1355 | 39.7±2.9 | 60.3±2.9 | 18.3 |
| mGL-Toc34WT + GMP-PNP | 71.9±6.5 | 28.1±6.5 | 1173 | 62.1±2.4 | 37.9±2.4 | 9.8 |
| mGL-Toc34WT + GTP-γ-S | 74.9±3.6 | 25.1±3.6 | 3195 | 57.4±5.8 | 42.6±5.8 | 10.3 |
| mGL-Toc34WT + SS-tp | 69.9±5.5 | 30.1±5.5 | 3176 | 63.7±11.3 | 36.3±11.3 | 6.2 |
| mGL-Toc34WT + RCMLa | 67.5±2.2 | 32.5±2.2 | 3135 | 41.5±2.1 | 58.5±2.1 | 27.0 |
| mEGFP-Toc34WT | 51.0±6.0 | 49.0±6.0 | 2902 | 64.1±4.3 | 35.9±4.3 | 25.4 |
| mEGFP-Toc34(R133A) | 82.4±5.0 | 17.6±5.0 | 2227 | 89.2±8.2 | 10.8±8.2 | 10.9 |
| mEGFP-Toc34WT + GTP | 56.5±2.4 | 43.5±2.4 | 928 | 64.1±3.5 | 35.9±3.5 | 7.6 |
| mEGFP-Toc34WT + GMP-PNP | 84.5±3.1 | 15.5±3.1 | 1323 | 89.7±2.0 | 10.3±2.0 | 5.2 |
| mEGFP-Toc34WT + GTP-γ-S | 77.0±2.5 | 23.0±2.5 | 1307 | 79.3±13.6 | 20.7±13.6 | 4.3 |
| mEGFP-Toc34WT + SS-tp | 90.4±1.8 | 9.6±1.8 | 1773 | 86.6±7.6 | 13.4±7.6 | 3.8 |
| mEGFP-Toc34WT + RCMLa | 67.0±5.1 | 33.0±5.1 | 4520 | 77.5±7.3 | 22.5±7.3 | 10.5 |

**Table S2. Table showing the population distribution of monomers and dimers as measured by single-molecule and crosslinking assays.** All the sample conditions tested are mentioned in the first column, followed by monomer and dimer percentages obtained via single-molecule detection. n is the number of traces quantified to determine percent monomers and dimers. Quantifications obtained from immunoblots of crosslinking data are presented in the subsequent columns, followed by the percentage difference between single-molecule and crosslinking data.

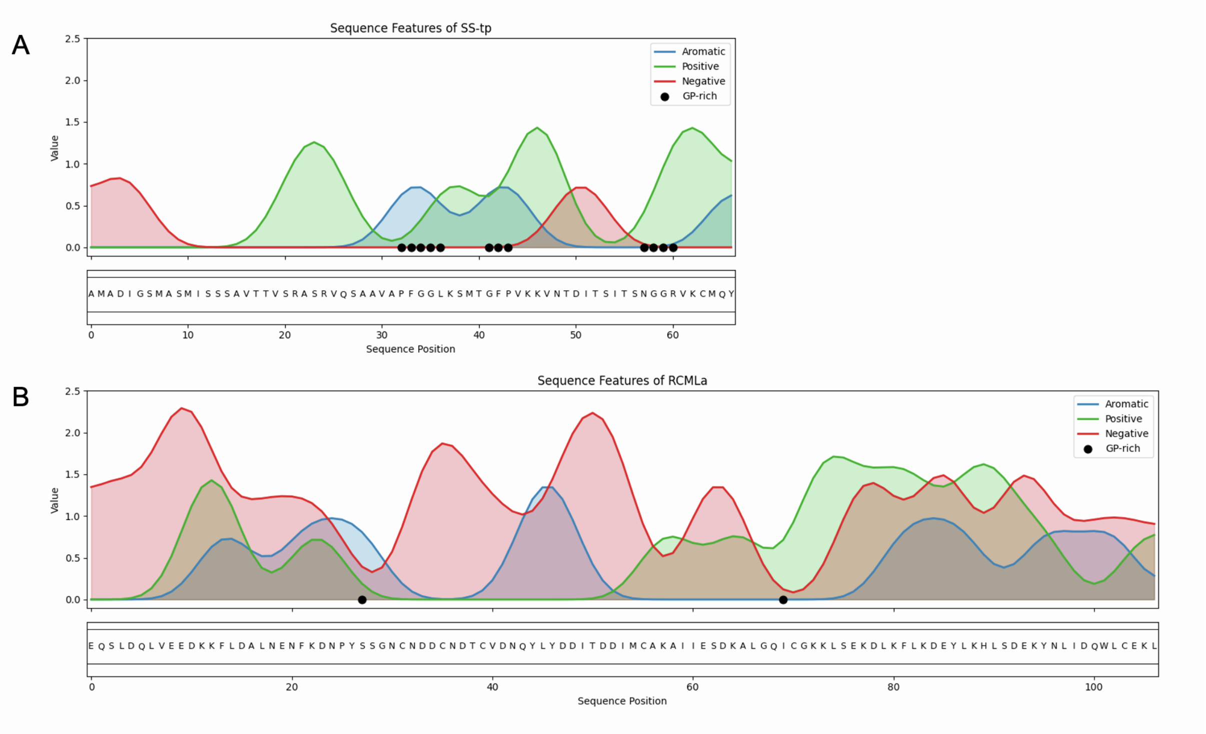


**Figure. S8 Features of SS-tp and RCMLa involved in Toc34 recognition.** (*A)* A feature plot of SS-tp showing its aromatic residues in blue, positive residues in green, negative residues in red, and G/P rich sites in black dots on y=0. The bottom panel shows the SS-tp sequence and its position aligned with its feature plot. (*B)* A feature plot of RCMLa showing its aromatic residues in blue, positive residues in green, negative residues in red, and G/P rich sites in black dots on y=0. The bottom panel shows the RCMLa sequence and its position aligned with its feature plot.

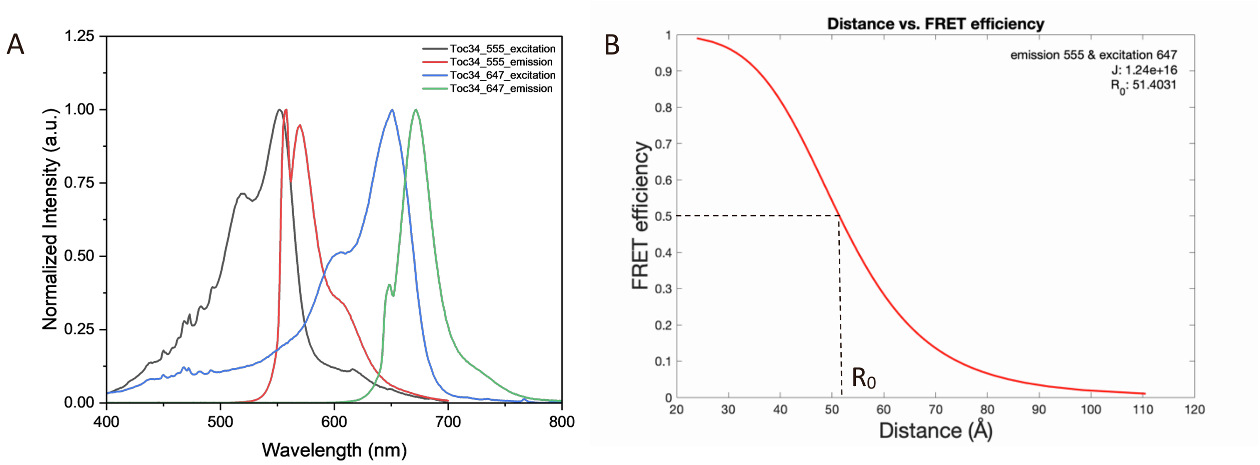


C

| FRET efficiency, E | Distance, $r_0$ (Å) |
|---|---|
| 0.42 | 54.24 |
| 0.65 | 46.36 |
| 0.85 | 38.50 |

**Figure S9. Characterization of labelled Toc34.** (*A)* Excitation and emission spectra of Alexa 555- and Alexa 647-labelled Toc34. (*B)* Distance vs FRET efficiency plot to determine the distance between the fluorophores at different FRET states. *(C)* A table showing calculated distances for respective FRET states.

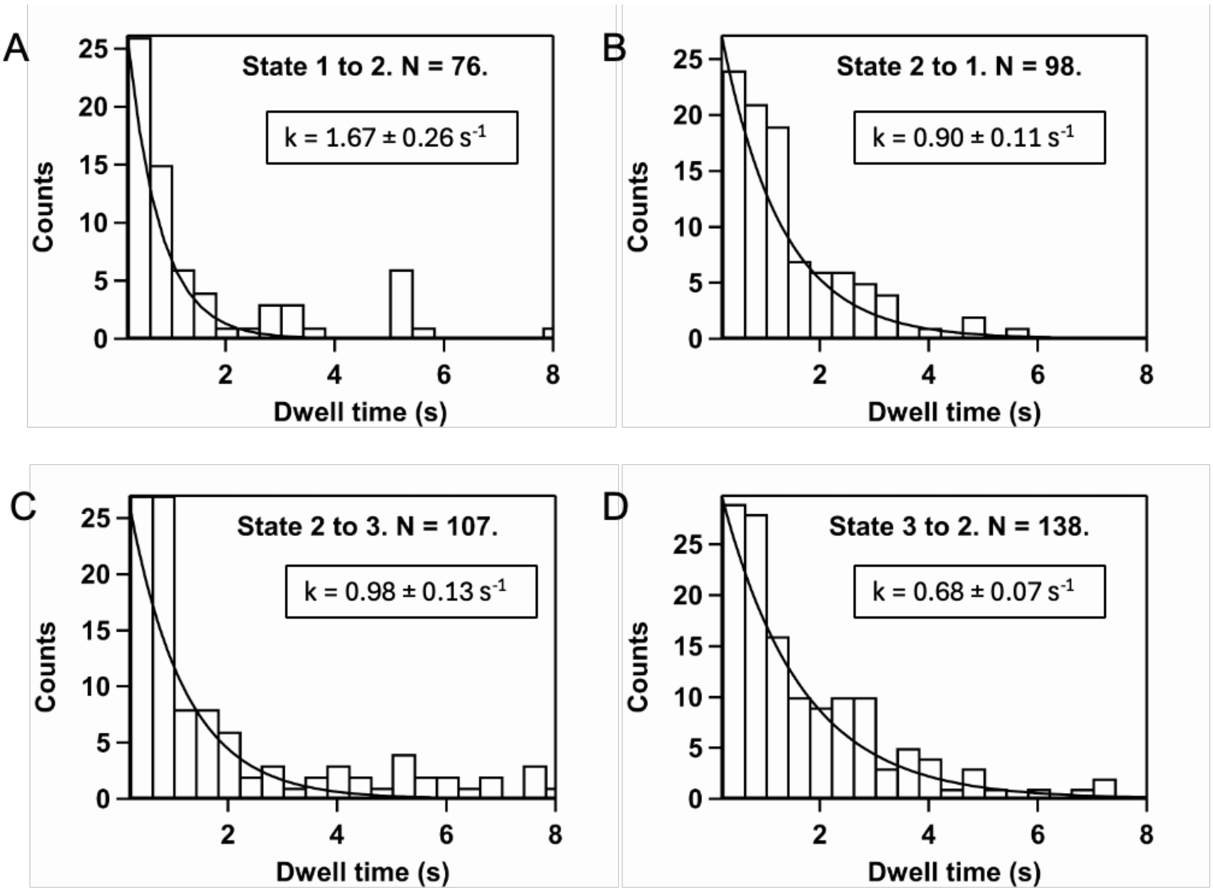


**Figure S10. Histogram of dwell times at different transitions.** (*A)* Histogram of population transitioning from FRET state 0.42 to 0.65. (*B)* Histogram of population transitioning from FRET state 0.65 to 0.42. (*,)* Histogram of population transitioning from FRET state 0.65 to 0.85. (*D*) Histogram of population transitioning from FRET state 0.85 to 0.65. All histograms are fitted with a single-exponential decay, with the slope providing the rate constant (k).

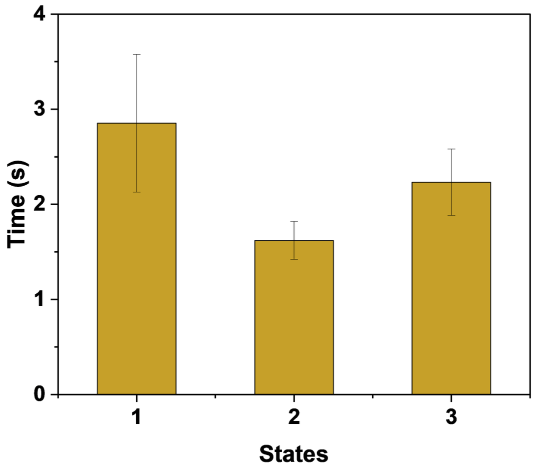


**Figure S11. Mean occupancies at different FRET States.** A bar plot of mean occupancy times with their respective standard deviation at FRET states 1 (0.42), 2 (0.65), and 3 (0.85).

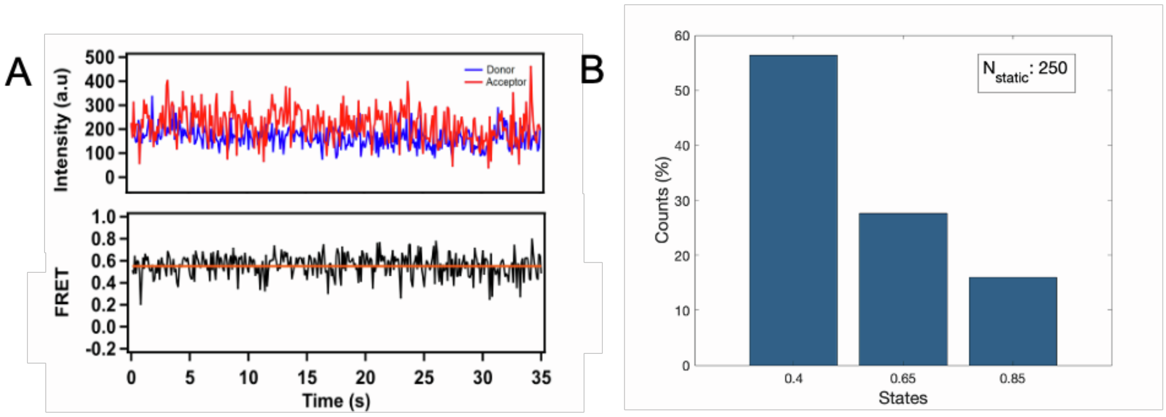


**Figure S12. Static population of Toc34 bound to GDP.** (*A)* A representative static single-molecule fluorescence intensity time trace. The top panel shows the donor in blue and acceptor in red, and the bottom panel shows the corresponding FRET trace in black with its HMM fit in orange. (*B)* bar plot showing the percent count of static traces at 0.4, 0.65, and 0.85 FRET states.

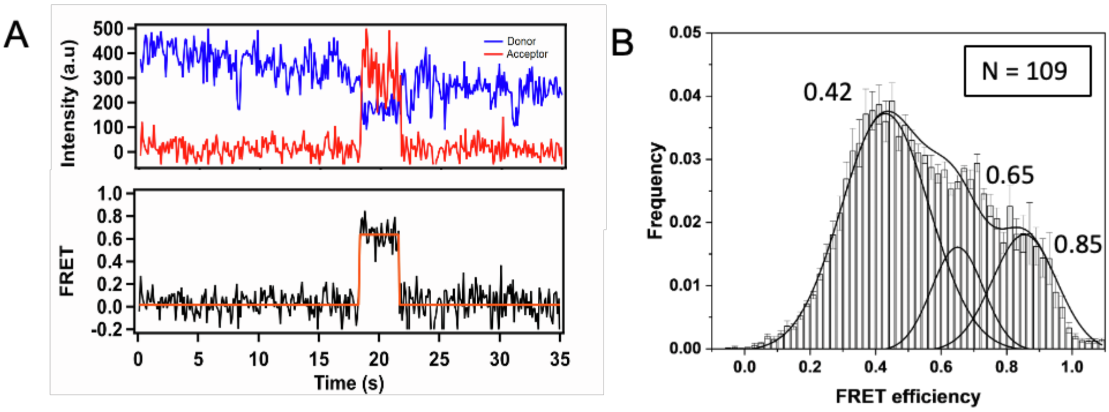


**Figure. S13 Transient of Toc34 dimer observed in its GDP-bound state**. (*A)* A representative transient single-molecule fluorescence intensity time trace. The top panel shows the donor in blue and acceptor in red, and the bottom panel shows the corresponding FRET trace in black with its HMM fit in orange. (*B)* Histogram of all transient traces with Gaussian fitting at FRET states at 0.42, 0.65, and 0.85.

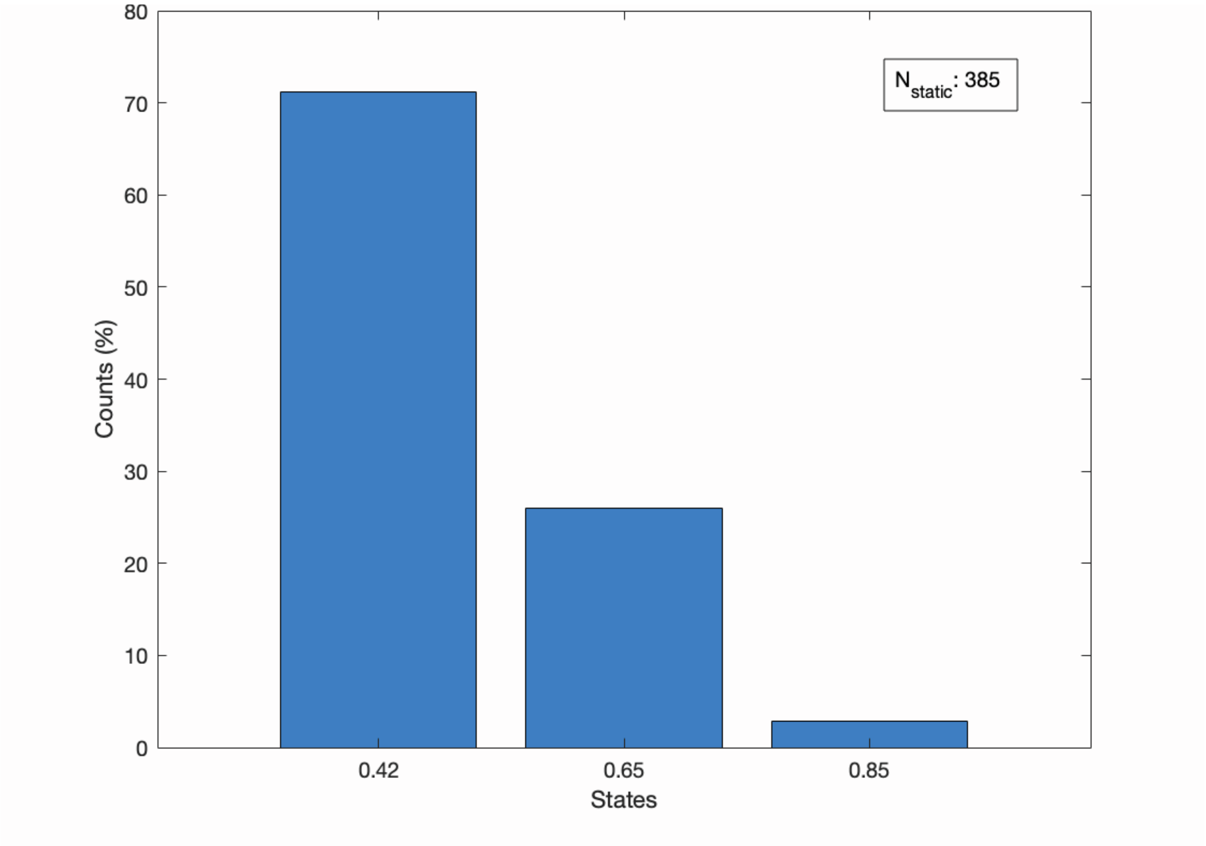


**Figure S14. Static population of Toc34 bound to RCMLa.** A bar plot showing the percent count of static traces at 0.42, 0.65, and 0.85 FRET states.

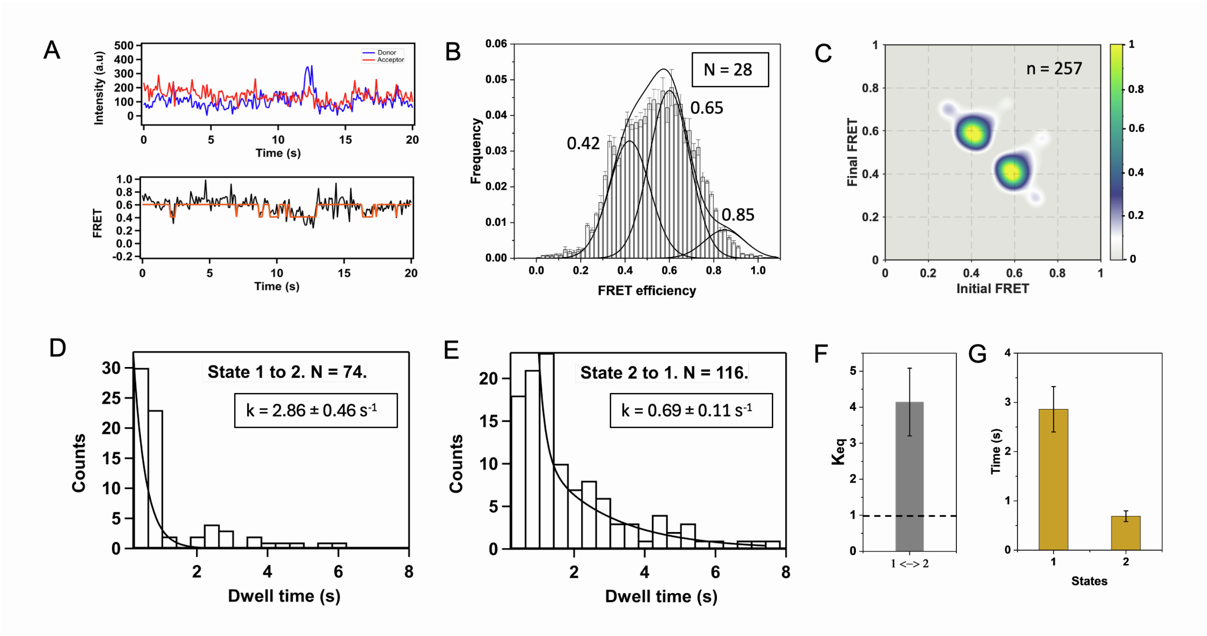


**Figure. S15 Dynamics of Toc34 homodimer in the presence of disordered peptide, RCMLa.** (*A)* A representative dynamic single-molecule fluorescence intensity time trace. The top panel shows the donor in blue and acceptor in red, and the bottom panel shows the corresponding FRET trace in black with its HMM fit in orange. (*B)* Histogram of all dynamic traces with Gaussian fitting, showing FRET states at 0.42, 0.65, and 0.85. (*C)* A transition density plot (TDP) showing a heat map of all transitions (n) observed among the dynamic traces, and (*D)* Histogram of population transitioning from FRET state 0.42 to 0.65, fitted in a single exponential decay. € Histogram of population transitioning from FRET state 0.65 to 0.42, fitted in a single exponential decay. (*F)* A bar plot showing equilibrium constant (Keq) and the standard deviation for the transition between state 1 and 2, and (*G)* A bar plot showing mean occupancy times with their respective standard deviation at FRET states 1 (0.42) and 2 (0.65).
